# Supplementary material for: Role of community health workers in improving cost efficiency in an active case finding tuberculosis programme: an operational research study from rural Bihar, India
Source: BMJ Open. 2020 Oct 1;10(10):e036625. doi: 10.1136/bmjopen-2019-036625 (PMC7536783; doi:10.1136/bmjopen-2019-036625)
Supplement: Supplementary data [file bmjopen-2019-036625supp001.pdf]

Supplementary File 1: The cost breakdown of the active case-finding program from Q3 2017 to Q2 2018 with cost allocation between case-finding and treatment support activities

									Cost allocation		Case-finding costs proportionate to the cost allocation				
**#**	**Category**	**Item**	**2017Q3**	**2017Q4**	**2018Q1**	**2018Q2**	**Total**	**Comment**	**Case-finding**	**Treatment**	**C2017Q3**	**C2017Q4**	**C2018Q1**	**C2018Q2**	**Ctotal**
1	Activities	Trainers, per-diem costs	₹ 29,850	₹ 29,850	₹ 29,850	₹ 29,850	₹ 1,19,400		100%	0%	₹ 29,850	₹ 29,850	₹ 29,850	₹ 29,850	₹ 1,19,400
2	Activities	Case-finding incentives	₹ 1,39,500	₹ 1,48,000	₹ 1,57,000	₹ 1,52,500	₹ 5,97,000	Unit cost INR500	100%	0%	₹ 1,39,500	₹ 1,48,000	₹ 1,57,000	₹ 1,52,500	₹ 5,97,000
3	Activities	Treatment completion incentives	₹ 2,65,050	₹ 2,81,200	₹ 2,98,300	₹ 2,89,750	₹ 11,34,300	Unit cost INR1000	0%	100%	₹ -	₹ -	₹ -	₹ -	₹ -
4	Activities	Field visits (fuel costs, management)	₹ 2,71,541	₹ 3,11,828	₹ 3,69,122	₹ 3,23,806	₹ 12,76,297		66%	34%	₹ 1,80,029	₹ 2,06,739	₹ 2,44,724	₹ 2,14,680	₹ 8,46,171
5	Activities	Training and workshops	₹ 65,395	₹ 52,488	₹ 58,690	₹ 2,69,343	₹ 4,45,916		55%	45%	₹ 35,925	₹ 28,835	₹ 32,242	₹ 1,47,965	₹ 2,44,967
6	Activities	Transport allowance	₹ 24,059	₹ 73,053	₹ 1,06,012	₹ 1,04,381	₹ 3,07,505		100%	0%	₹ 24,059	₹ 73,053	₹ 1,06,012	₹ 1,04,381	₹ 3,07,505
7	Activities	Communication material	₹ 24,474	₹ 15,400	₹ 50,265	₹ 16,950	₹ 1,07,089		100%	0%	₹ 24,474	₹ 15,400	₹ 50,265	₹ 16,950	₹ 1,07,089
8	Administrative overheads	Information technology (mobile data, voice)	₹ 25,020	₹ 25,638	₹ 16,969	₹ 13,150	₹ 80,777		66%	34%	₹ 16,588	₹ 16,998	₹ 11,250	₹ 8,718	₹ 53,554
9	Administrative overheads	Car rental (per month)	₹ 1,50,000	₹ 1,50,000	₹ 1,50,000	₹ 1,50,000	₹ 6,00,000		81%	19%	₹ 1,21,835	₹ 1,21,835	₹ 1,21,835	₹ 1,21,835	₹ 4,87,339
10	Administrative overheads	Rent	₹ 43,900	₹ 51,900	₹ 51,900	₹ 51,900	₹ 1,99,600		81%	19%	₹ 35,657	₹ 42,155	₹ 42,155	₹ 42,155	₹ 1,62,121
11	Administrative overheads	Electricity	₹ 4,085	₹ 8,055	₹ 7,740	₹ 7,654	₹ 27,534		81%	19%	₹ 3,318	₹ 6,543	₹ 6,287	₹ 6,217	₹ 22,364
12	Administrative overheads	Supplies (stationery, workshops, etc.)	₹ 1,93,094	₹ 1,10,390	₹ 88,210	₹ 78,000	₹ 4,69,694		81%	19%	₹ 1,56,837	₹ 89,662	₹ 71,647	₹ 63,354	₹ 3,81,500
13	Human resources	Program manager	₹ 2,10,000	₹ 2,10,000	₹ 2,10,000	₹ 2,10,000	₹ 8,40,000		81%	19%	₹ 1,70,569	₹ 1,70,569	₹ 1,70,569	₹ 1,70,569	₹ 6,82,275
14	Human resources	Project manager (community and training)	₹ 1,35,000	₹ 1,35,000	₹ 1,35,000	₹ 2,03,226	₹ 6,08,226		81%	19%	₹ 1,09,651	₹ 1,09,651	₹ 1,09,651	₹ 1,65,067	₹ 4,94,020
15	Human resources	Project manager (service delivery)	₹ 2,10,484	₹ 2,70,000	₹ 1,46,613	₹ 1,35,000	₹ 7,62,097		81%	19%	₹ 1,70,962	₹ 2,19,303	₹ 1,19,084	₹ 1,09,651	₹ 6,18,999
16	Human resources	GeneXpert technician	₹ 56,903	₹ 1,02,000	₹ 1,02,000	₹ 1,02,000	₹ 3,62,903		100%	0%	₹ 56,903	₹ 1,02,000	₹ 1,02,000	₹ 1,02,000	₹ 3,62,903
17	Human resources	MIS operator	₹ 49,484	₹ 90,250	₹ 1,01,000	₹ 1,16,250	₹ 3,56,984		66%	34%	₹ 32,807	₹ 59,835	₹ 66,962	₹ 77,073	₹ 2,36,677
18	Human resources	Consultants	₹ 1,66,833	₹ 1,65,000	₹ -	₹ -	₹ 3,31,833		100%	0%	₹ 1,66,833	₹ 1,65,000	₹ -	₹ -	₹ 3,31,833
19	Human resources	Block coordinators (BCs)	₹ 1,92,000	₹ 1,92,000	₹ 1,92,000	₹ 1,92,000	₹ 7,68,000		62%	38%	₹ 1,19,897	₹ 1,19,897	₹ 1,19,897	₹ 1,19,897	₹ 4,79,588
20	Human resources	Field coordinators (FCs)	₹ 4,11,196	₹ 4,02,155	₹ 4,48,906	₹ 4,59,767	₹ 17,22,024		55%	45%	₹ 2,25,893	₹ 2,20,926	₹ 2,46,609	₹ 2,52,576	₹ 9,46,005
21	Procurement of medical items	GeneXpert, test cartridge	₹ 1,29,006	₹ 6,26,923	₹ 6,20,888	₹ 3,39,489	₹ 17,16,306	Unit cost USD11.26	100%	0%	₹ 1,29,006	₹ 6,26,923	₹ 6,20,888	₹ 3,39,489	₹ 17,16,306
22	Procurement of medical items	Contingency, drugs	₹ 38,127	₹ 98,864	₹ 1,28,695	₹ 1,65,859	₹ 4,31,545		0%	100%	₹ -	₹ -	₹ -	₹ -	₹ -
23	Procurement of medical items	Contingency, chest X-rays	₹ 1,32,823	₹ 2,57,945	₹ 3,39,280	₹ 2,11,545	₹ 9,41,593		100%	0%	₹ 1,32,823	₹ 2,57,945	₹ 3,39,280	₹ 2,11,545	₹ 9,41,593
24	Procurement of medical items	Contingency, sputum microscopy	₹ 11,280	₹ 3,980	₹ 13,400	₹ 9,200	₹ 37,860		100%	0%	₹ 11,280	₹ 3,980	₹ 13,400	₹ 9,200	₹ 37,860
25	Procurement of medical items	Extra-pulmonary TB diagnostics	₹ 10,986	₹ 33,907	₹ 73,800	₹ 69,324	₹ 1,88,017		100%	0%	₹ 10,986	₹ 33,907	₹ 73,800	₹ 69,324	₹ 1,88,017
26	Procurement of medical items	Complications and hospitalization	₹ 25,719	₹ 45,597	₹ 42,386	₹ 36,111	₹ 1,49,813		0%	100%	₹ -	₹ -	₹ -	₹ -	₹ -
27	Procurement of medical items	Sputum containers (for transport)	₹ 8,894	₹ -	₹ -	₹ -	₹ 8,894		100%	0%	₹ 8,894	₹ -	₹ -	₹ -	₹ 8,894
28	Procurement of medical items	Customs duty and Xpert shipping	₹ 53,694	₹ 2,60,934	₹ 2,58,422	₹ 1,41,300	₹ 7,14,350	Unit cost INR314	100%	0%	₹ 53,694	₹ 2,60,934	₹ 2,58,422	₹ 1,41,300	₹ 7,14,350
29		*Additional information*													
30		Cartridges used	171	831	823	450									
31		Total TB diagnosed	284	302	324	326									
32		Total TB treatments started	279	296	314	305		See patient cascade in the figure							
33		Total treatment completed	265	281	298	290		Assumed at 95%							

* All cost figures are reported in Indian Rupee (INR or ₹)
* The cost allocation is sourced from the time calculations in Supplementary File 2
* The case-finding incentive (item 2) is derived from total cases diagnosed (item 31)
* The treatment completion incentive (item 3) is derived from total treatment completed (item 33)
* The GeneXpert costs (item 21 and 28) are derived from the actual consumption of the cartridges (item 30)
