# Supplementary material for: Role of community health workers in improving cost efficiency in an active case finding tuberculosis programme: an operational research study from rural Bihar, India
Source: BMJ Open. 2020 Oct 1;10(10):e036625. doi: 10.1136/bmjopen-2019-036625 (PMC7536783; doi:10.1136/bmjopen-2019-036625)
Supplement: Supplementary data [file bmjopen-2019-036625supp002.pdf]

Supplementary File 2: The time allocation of human resource between case-finding and treatment support activities

Table A: Detailed time allocation for human resource

#	Human Resource	Activities	Value	Comment
1	**Field coordinator (FC)**	Work hours (in hours)	48	6 working days, 8 hours a day; 5 day field work + 1 day reporting-review-planning-training
		Total patients diagnosed per month	105	100 - 110 patients diagnosed per month
		Average treatment duration per patient (in months)	7	DSTB treatment duration of 6 - 8 months
		Patients per field coordinator, steady state	35	18 field coordinators in the program
		Travel per week (in hours)	193	4 weeks in a month. Average travel reimbursement per field coordinator of ₹2700 at ₹3.5 per km.
1.1		**Travel time per week (in hours)**	10	Average speed of 20 kmph
1.2		**Treatment support activities**		
		Time per visit (in hours)	1	Half hour on average
		Number of visits per patient in a month	3	3 times a month
		Total time spent per week in treatment support activities (in hours)	13	
1.3		**Case-finding activities**		
		Time spent in ASHA (CHW) training per week (in hours)	2	Average 1 meeting per week lasting nearly 2 hour
		Time spent in meeting individual ASHAs per week (in hours)	2	4 ASHAs per week, 0.5 hour per meeting
		Time spent in hospital visits and assisting in diagnosis per week (in hours)	6	3 times a week, 2 hours per visit
		Time spent in meeting referrals and screening per week (in hours)	6	12 per week, 0.5 hour per visit
		Total time spent per week in case-finding activities (in hours)	16	
1.4		**Reporting-review-planning-training, time spent per week (in hours)**	8	1 day
2	**Block coordinator (BC)**	Work hours (in hours)	48	6 working days, 8 hours a day
2.1		Admin-reporting-review-planning, time spent per week (in hours)	8	1 complete day, focused around FCs in their area
2.2		Block review-data management-project meeting-training, time spent per week (in hours)	8	Project meeting once a week, data management with MIS team, reviewing project performance, training
2.3		Supervisory work, time spent per week (in hours)	32	Split in proportion to FCs time spent in activities
3	**Managers**	Work hours	48	6 working days, 8 hours a day
3.1		Review-planning-design, time spent per week (in hours)	24	3 days a week, largely centered around case-finding
3.2		Supervisory work, time spent per week (in hours)	24	Split in proportion to BCs time spent in activities
4	**Data management team**	Work hours	48	Split in proportion of FCs time under reporting-review-planning

Table B: Summary of time allocation between case-finding and treatment support activities

Field coordinator (FC)	Activity	Time	Travel	Reporting-review-planning-training	Total	Proportion
1	Case-finding activities	16.0	5.3	4.4	25.7	55%
2	Treatment support activities	13.1	4.3	3.6	21.1	45%

Note: Time spent in travel and reporting-review-planning-training is proportionate to time spent in respective case-finding and treatment activities.

Block coordinator (BC)	Activity	Admin-reporting-review-planning	Block review-data management-project meeting-training	Supervisory work, time spent per week	Proportion
1	Case-finding activities	4.4	8.0	17.6	62%
2	Treatment support activities	3.6	0.0	14.4	38%

Managers	Activity	Review-planning-design	Supervisory work	Proportion
1	Case-finding activities	24	15.0	81%
2	Treatment support activities	0	9.0	19%

Data management team	Activity	Time	Proportion
1	Case-finding activities	94.7	66%
2	Treatment support activities	48.1	34%

Table C: Proportion of time allocation for human resource

#	Human Resource	Case-finding	Treatment support
1	Field coordinator (FC)	55%	45%
2	Block coordinator (BC)	62%	38%
3	Managers	81%	19%
4	Data management team	66%	34%
