# Supplementary material for: Role of community health workers in improving cost efficiency in an active case finding tuberculosis programme: an operational research study from rural Bihar, India
Source: BMJ Open. 2020 Oct 1;10(10):e036625. doi: 10.1136/bmjopen-2019-036625 (PMC7536783; doi:10.1136/bmjopen-2019-036625)
Supplement: Supplementary data [file bmjopen-2019-036625supp003.pdf]

Supplementary File 3: Additional information on ASHA

Table A: Number of ASHAs

#	Block	Number of ASHA^	Population	ASHAs per 1000 pop.
1	Ujiarpur	264	341906	0.77
2	Saraianjan	236	287760	0.82
3	Bibhutipur	293	391817	0.75
	Total	793	1021483	0.78

Note: ^ Data as of December 2017.

Table B: Work of FC and ASHAs

Indicator	Steady state	Maximum
Total FC	18	23
Pop. per FC	56749	44412
ASHA per FC	44	34
Intervention pop.	1021483	

Note:

1. FC is Field Coordinator

2. Steady state inicates matured program operations and maximum indicate their highest value during study period.

Table C1: ASHA's performance on reproductive, maternal, and child health program indicators in Samastipur, Bihar

		Baseline period				Implementation period				Quarterly average	
Indicator	Region	2016Q3	2016Q4	2017Q1	2017Q2	2017Q3	2017Q4	2018Q1	2018Q2	Baseline	Implementation
Total number of pregnant women registered for ANC	IR	5947	5137	6330	6229	6415	5895	6593	6178	5911	6270
	CR	5951	5333	6340	6769	6387	6083	6648	6190	6098	6327
Number of Institutional Deliveries conducted (Including C-Sections)	IR	4592	4116	3913	3225	5011	4419	3694	3134	3962	4065
	CR	4045	3646	3809	2739	4482	4015	3487	2631	3560	3654
Number of Immunisation sessions where ASHAs were present	IR	2528	2531	2535	2606	2528	2550	2574	2566	2550	2555
	CR	2743	2728	2719	2673	2583	2626	2674	2672	2716	2639

Table C2: Detailed disaggregation of data in Table C1

		Baseline period				Implementation period				
Indicator	Region	2016Q3	2016Q4	2017Q1	2017Q2	2017Q3	2017Q4	2018Q1	2018Q2	Block
Total number of pregnant women registered for ANC	IR	2312	1788	2376	2278	2323	1832	2267	2296	Bibhutipur
	IR	1562	1510	1858	1980	1959	1899	2169	1993	Sarairanjan
	IR	2073	1839	2096	1971	2133	2164	2157	1889	Ujiarpur
	CR	2157	1837	2461	2413	2278	2040	2477	2095	Kalyanpur
	CR	802	659	904	792	929	891	911	804	Pusa
	CR	1433	1220	1341	1714	1645	1761	1512	1552	Singhia
	CR	1559	1617	1634	1850	1535	1391	1748	1739	Warisnagar
Number of Institutional Deliveries conducted (Including C-Sections)	IR	1562	1501	1396	1125	1710	1531	1354	1180	Bibhutipur
	IR	1676	1458	1392	1157	1827	1605	1326	1063	Sarairanjan
	IR	1354	1157	1125	943	1474	1283	1014	891	Ujiarpur
	CR	1059	931	1093	725	1160	867	938	801	Kalyanpur
	CR	816	675	661	546	833	716	630	502	Pusa
	CR	995	995	1033	671	1186	1264	986	639	Singhia
	CR	1175	1045	1022	797	1303	1168	933	689	Warisnagar
Number of Immunisation sessions where ASHAs were present	IR	923	909	915	933	931	907	927	928	Bibhutipur
	IR	736	765	763	800	768	767	772	773	Sarairanjan
	IR	869	857	857	873	829	876	875	865	Ujiarpur
	CR	995	956	976	982	969	964	980	971	Kalyanpur
	CR	424	445	412	388	363	363	373	378	Pusa
	CR	606	622	628	631	588	617	615	614	Singhia
	CR	718	705	703	672	663	682	706	709	Warisnagar

Note:

1. This is the dataset used for Table 3 in the manuscript.

2. Baseline period: Q3 of 2016 to Q2 of 2017

3. Study period: Q3 of 2017 to Q2 of 2018

4. IR: Intervention region

5. CR: Control region

6. ASHA: Accredited Social Health Activist

7. ANC: Antenatal checkup
