# Supplementary material for: Role of community health workers in improving cost efficiency in an active case finding tuberculosis programme: an operational research study from rural Bihar, India
Source: BMJ Open. 2020 Oct 1;10(10):e036625. doi: 10.1136/bmjopen-2019-036625 (PMC7536783; doi:10.1136/bmjopen-2019-036625)
Supplement: Supplementary data [file bmjopen-2019-036625supp004.pdf]

Supplementary File 4: The disaggregated yield of the active case-finding program from Q3 2017 to Q2 2018.

Table A: Summary of yield by various blocks

#	Indicator	Total	Bibhutipur	Sarairanjan	Ujiarpur
1	# of people eligible for screening (referrals)	12394	4702	3764	3928
2	# of people screened	11233	4175	3403	3655
3	# of people with TB symptoms	9895	3573	3066	3256
4	# of people tested/evaluated for TB	5864	2062	1870	1932
5	# of people diagnosed with TB	1236	439	461	336
6	# of people initiated on treatment	1194	427	437	330
7	# of people diagnosed with TB (Bac+)	637	167	253	217
8	Proportion of microbiologically diagnosed cases	52%	38%	55%	65%
9	Pre-diagnostic loss to follow-up	41%	42%	39%	41%
10	Pre-treatment loss to follow-up	3%	3%	5%	2%
11	% screened of referred	91%	89%	90%	93%
12	% presumptive of screened	88%	86%	90%	89%
13	% tested of presumptive	59%	58%	61%	59%
14	% diagnosed with TB of tested	21%	21%	25%	17%

Note: Bac+ means microbiologically-confirmed TB.

Table B: Details of yield by various blocks and quarters

		Bibhutipur				Sarairanjan				Ujiarpur				Overall			
**#**	**Indicator**	**B 17Q3**	**B 17Q4**	**B 18Q1**	**B 18Q2**	**S 17Q3**	**S 17Q4**	**S 18Q1**	**S 8Q2**	**U 17Q3**	**U 17Q4**	**U 18Q1**	**U 18Q2**	**17Q3**	**17Q4**	**18Q1**	**18Q2**
1	# of people eligible for screening (referrals)	830	1320	1513	1039	794	781	988	1201	678	1080	1126	1044	2302	3181	3627	3284
2	# of people screened	698	1135	1420	922	648	737	933	1085	618	974	1078	985	1964	2846	3431	2992
3	# of people with TB symptoms	495	983	1273	822	593	662	870	941	502	894	983	877	1590	2539	3126	2640
4	# of people tested/evaluated for TB	251	566	762	483	334	383	574	579	276	519	597	540	861	1468	1933	1602
5	# of people diagnosed with TB	83	102	136	118	121	114	114	112	80	86	74	96	284	302	324	326
6	# of people initiated on treatment	83	100	132	112	117	111	109	100	79	85	73	93	279	296	314	305
7	# of people diagnosed with TB (Bac+)	32	45	53	37	50	51	78	74	55	48	53	61	137	144	184	172
8	Proportion of microbiologically diagnosed cases	39%	44%	39%	31%	41%	45%	68%	66%	69%	56%	72%	64%	48%	48%	57%	53%
9	Pre-diagnostic loss to follow-up	49%	42%	40%	41%	44%	42%	34%	38%	45%	42%	39%	38%	46%	42%	38%	39%
10	Pre-treatment loss to follow-up	0%	2%	3%	5%	3%	3%	4%	11%	1%	1%	1%	3%	2%	2%	3%	6%
11	% screened of referred	84%	86%	94%	89%	82%	94%	94%	90%	91%	90%	96%	94%	85%	89%	95%	91%
12	% presumptive of screened	71%	87%	90%	89%	92%	90%	93%	87%	81%	92%	91%	89%	81%	89%	91%	88%
13	% tested of presumptive	51%	58%	60%	59%	56%	58%	66%	62%	55%	58%	61%	62%	54%	58%	62%	61%
14	% diagnosed with TB of tested	33%	18%	18%	24%	36%	30%	20%	19%	29%	17%	12%	18%	33%	21%	17%	20%
		Bibhutipur				Sarairanjan				Ujiarpur				Overall			

Note:

1. B: Bibhutipur

2. S: Sarairanjan

3. U: Ujiarpur
