# Supplementary material for: Role of community health workers in improving cost efficiency in an active case finding tuberculosis programme: an operational research study from rural Bihar, India
Source: BMJ Open. 2020 Oct 1;10(10):e036625. doi: 10.1136/bmjopen-2019-036625 (PMC7536783; doi:10.1136/bmjopen-2019-036625)
Supplement: Author's manuscript [file bmjopen-2019-036625.draft_revisions.pdf]

BMJ Open is committed to open peer review. As part of this commitment we make the peer review history of every article we publish publicly available.

When an article is published we post the peer reviewers' comments and the authors' responses online. We also post the versions of the paper that were used during peer review. These are the versions that the peer review comments apply to.

The versions of the paper that follow are the versions that were submitted during the peer review process. They are not the versions of record or the final published versions. They should not be cited or distributed as the published version of this manuscript.

BMJ Open is an open access journal and the full, final, typeset and author-corrected version of record of the manuscript is available on our site with no access controls, subscription charges or pay-per-view fees (http://bmjopen.bmj.com).

If you have any questions on BMJ Open's open peer review process please email info.bmjopen@bmj.com

BMJ Open

Leveraging community health workers for active case finding in rural India: outcomes and costs of a novel TB initiative

Journal:	*BMJ Open*
Manuscript ID	bmjopen-2019-036625
Article Type:	Original research
Date Submitted by the Author:	22-Dec-2019
Complete List of Authors:	Garg, Tushar; Innovators In Health, Research<br>Bhardwaj, Manish; Innovators In Health, Operations<br>Deo, Sarang; Indian School of Business, Operations Management
Keywords:	Public health < INFECTIOUS DISEASES, Tuberculosis < INFECTIOUS DISEASES, PUBLIC HEALTH, Health economics < HEALTH SERVICES ADMINISTRATION & MANAGEMENT, International health services < HEALTH SERVICES ADMINISTRATION & MANAGEMENT, Organisation of health services < HEALTH SERVICES ADMINISTRATION & MANAGEMENT

SCHOLARONE™
Manuscripts

I, the Submitting Author has the right to grant and does grant on behalf of all authors of the Work (as defined in the below author licence), an exclusive licence and/or a non-exclusive licence for contributions from authors who are: i) UK Crown employees; ii) where BMJ has agreed a CC-BY licence shall apply, and/or iii) in accordance with the terms applicable for US Federal Government officers or employees acting as part of their official duties; on a worldwide, perpetual, irrevocable, royalty-free basis to BMJ Publishing Group Ltd ("BMJ") its licensees and where the relevant Journal is co-owned by BMJ to the co-owners of the Journal, to publish the Work in this journal and any other BMJ products and to exploit all rights, as set out in our licence.

The Submitting Author accepts and understands that any supply made under these terms is made by BMJ to the Submitting Author unless you are acting as an employee on behalf of your employer or a postgraduate student of an affiliated institution which is paying any applicable article publishing charge ("APC") for Open Access articles. Where the Submitting Author wishes to make the Work available on an Open Access basis (and intends to pay the relevant APC), the terms of reuse of such Open Access shall be governed by a Creative Commons licence – details of these licences and which Creative Commons licence will apply to this Work are set out in our licence referred to above.

Other than as permitted in any relevant BMJ Author's Self Archiving Policies, I confirm this Work has not been accepted for publication elsewhere, is not being considered for publication elsewhere and does not duplicate material already published. I confirm all authors consent to publication of this Work and authorise the granting of this licence.

For peer review only

TITLE

Leveraging community health workers for active case finding in rural India: outcomes and costs of a novel TB initiative

MANUSCRIPT TYPE

Research article

AUTHORS

Tushar Garg[1], Manish Bhardwaj[1], Sarang Deo[2]

[1] Innovators In Health, Patna

[2] Indian School of Business, Hyderabad

ADDRESS FOR CORRESPONDENCE

Tushar Garg,

Innovators in Health, #176, Ground Floor, Road #2,

New Pataliputra Colony, Patna – 800013. Bihar. India.

Phone: +91 98286 09562

Email: tgarg@innovatorsinhealth.org

MANUSCRIPT DETAILS

Abstract: 294 words

Manuscript: 3001 words

Tables: 4

Figures: 4

KEYWORDS

Accredited Social Health Activist (ASHA), Community Health Workers, Tuberculosis Active Case Finding, Cost Analysis

For peer review only - http://bmjopen.bmj.com/site/about/guidelines.xhtml

ABSTRACT

objectives

Cost-efficient active case finding (ACF) approaches are needed for their large-scale adoption in national TB programs (NTP). Our aim was to assess if CHWs' knowledge about health statuses of families can improve cost-efficiency of ACF program without adversely affecting delivery of other health services for which they are responsible.

design

Quasi-experimental design

interventions

We evaluated an ACF program in Samastipur district in Bihar, India between May 2017 and June 2018. CHWs (locally known as "ASHA") generated referrals of individuals at risk of TB and conducted symptom-based screening to identify presumptive TB patients. They also helped them undergo testing and provided treatment support for confirmed TB cases

primary and secondary outcome measures

We compared the notification rate from the intervention region with that from a control region in the same district with similar characteristics. We analyzed operational data to calculate the cost per TB case diagnosed. We used routine programmatic data from the public health system to estimate the impact on other services provided by CHWs.

findings

CHWs identified 9884 presumptive TB patients. Of these, 5852 patients were tested for TB and 1224 were confirmed as TB cases. Annual public case notification rate increased sharply in the intervention area from 45.8 to 105.8 per 100,000 population whereas it decreased from 50.7 to 45.3 in the control region. There was no practically or statistically significant impact on other output indicators of the CHWs, such as institutional deliveries (-0.04%). The overall cost of the intervention was about USD135.4 per diagnosed case. The main cost drivers were human resources, and commodities (drugs and diagnostics), which contributed 37.4% and 32.5% of the cost, respectively.

conclusions

ACF programs that utilize existing CHWs in the health system are feasible, cost-efficient and do not adversely affect other healthcare services delivered by CHWs.

For peer review only - http://bmjopen.bmj.com/site/about/guidelines.xhtml

STRENGTHS AND LIMITATIONS OF THIS STUDY

1. A pragmatic ACF implementation which utilized existing CHWs in the health system.
2. Used a comparable control region to obtain the incremental effect of the intervention.
3. Purposively selection of areas, hence, not a randomized control trial.
4. Patient costs incurred or averted and the NTP costs not included.
5. Small scale of the study and geographical location limit generalizability

For peer review only

For peer review only - http://bmjopen.bmj.com/site/about/guidelines.xhtml

INTRODUCTION

The World Health Organization (WHO) estimates about 10 million people falling ill with TB and nearly 1.5 million dying of it in 2018.[1] Despite continuous increase in case notifications in recent years, the 2018 estimates predict a gap of as much as 30% between incident and notified cases globally. Progress toward WHO's target of 90% reduction in TB incidence rate by 2035 is severely limited by existing passive case-finding (PCF) approaches that wait for patients to seek care at a health facility.[2–4] As a result, these approaches fail to address significant barriers in accessing care such as poor geographical and financial access, stigma, and poor awareness.[5]

Active case-finding (ACF) can address these challenges by finding previously undetected cases, initiating them on treatment promptly and thereby decreasing TB incidence in the long run.[4,6–8] In contrast with passive approaches, ACF is a health system initiated screening process that uses context-specific diagnostic algorithms and accommodates various implementation strategies including mass radiography, contact investigation, and house-to-house surveys.[9–11] Although modelling studies have shown ACF strategies to be cost-effective, cost per diagnosed case of such programs can be very high thereby limiting their large-scale adoption.[12–15] As a result, there is limited empirical evidence from high-burden and resource-constrained settings to inform key operational decisions regarding ACF programs: who will conduct ACF activities, how will they be integrated within the health system, and how will these additional activities impact other health services.[16].

In this study, we address these questions with evidence from a novel intervention in rural India that leveraged existing Community Health Workers (CHWs) in the public health system for ACF activities. In particular, our aim is to assess if CHWs' knowledge about health statuses of families can improve cost-efficiency of ACF program without adversely affecting delivery of other health services for which they are responsible.

METHODS

study design

Our intervention was implemented from May 15th, 2017 with the approval of the state and district health administration as an extension of routine services provided as part of the Revised National TB Control Program (RNTCP). We used a quasi-experimental design to evaluate the impact of the intervention over a period between July 1st, 2017 and June 30th,

For peer review only - http://bmjopen.bmj.com/site/about/guidelines.xhtml

2018. We used aggregate intervention and programmatic data for our analysis and hence did not require ethics approval for the study.

study setting

Our study was conducted in the Samastipur district of the east Indian state of Bihar. In 2011, it had a population of about 42.6 million, of which 96.5% lived in rural areas. The literacy rate was 50.3% and the sex ratio was 911.[17] Main source of income in more than 71.3% households was casual labor and the highest individual income was less than INR5000 (USD71) in 69.1% households.[18] Total fertility rate in the district was 3.8 and infant mortality rate was 53 deaths per 1000 live births.[19] More than 70% births occurred at a healthcare institution.[20] In 2017, annual TB case notification rate for the district was 55 per 100,000 population with a pre-treatment loss to follow-up (PTLFU) rate of 25%. In 2016, successful treatment outcome was reported for 72% of the microbiologically confirmed (Bac+) new TB cases (44% of all cases).[21]

The intervention region (IR) consisted of three blocks—Ujiarpur, Bibhutipur, and Sarairanjan—with a total population of 1,021,483.[Figure 1]. We chose four blocks—Kalyanpur, Warisnagar, Pusa, and Singhia—as the control region (CR) with a population of 981,924.[22] These were geographically separated from the IR to minimize spill-over effects of the intervention.

IR and CR were similar along relevant sociodemographic variables such as proportion of population belonging to scheduled castes (18.2% vs. 20.8%).[18][Table 1] Further, the structure of the public health systems in IR and CR was comparable on relevant dimensions. Each block in IR as well as CR coincided with a Tuberculosis Unit (TU) under the Revised National TB Control Program (RNTCP), which was managed by a Senior Treatment Supervisor (STS). IR and CR included four designated microscopy centers (DMCs) each, where sputum microscopy was provided. Finally, the annual TB case notification rate was comparable across IR and CR (52 vs. 53.1 per 100,000 population in 2016).[23]

Table 1

The demographic characteristics of the intervention and control region in the active case-finding project

Characteristics	Intervention region	Control region

For peer review only - http://bmjopen.bmj.com/site/about/guidelines.xhtml

Blocks	3	4
Area (sq. km.)	582	623
Population	1021483	981924
Sex ratio	918	919
Proportion of scheduled castes population	18.2%	20.8%
Literacy rate	63.5%	59.8%
Households with monthly income of highest earning household member less than INR5000	69.8%	70.6%

intervention

We implemented an ACF intervention in collaboration with the RNTCP and the National Health Mission (NHM) with project funding from Stop TB Partnership's TB REACH. Under this intervention, we engaged with community health workers (CHWs), locally known as Accredited Social Health Activists (ASHAs), who work for the NHM. Their main role is community mobilization and facilitating last-mile delivery of health services across multiple programs though their focus is reproductive, maternal and child health. We trained these CHWs to identify patients with TB symptoms during their routine work and refer them to a field coordinator (FC). FCs further screened these patients using a symptom-based tool after obtaining their verbal consent.[9] Presumptive TB patients identified through screening were accompanied by ASHAs to the nearest PHC for diagnostic testing and physician consultation. All presumptive TB patients underwent sputum microscopy and chest X-ray (CXR). GeneXpert testing, if indicated by the diagnostic protocol, was conducted at the laboratory operated using project funding. Upon confirmation of TB diagnosis, ASHA obtained drugs from the STS and initiated treatment at patient's residence. For each confirmed case of TB, the project paid INR200 (USD3) to ASHA for referral, and INR300 (USD4.5) to ASHA for assisting in diagnosis and treatment initiation. ASHAs counselled patients on the importance of adherence and treatment completion and monitored them for adverse effects through regular follow-up household visits. They received INR400 (USD6) after their first follow-up visit and INR600 (USD9) upon successful completion of treatment.[Figure 2] In addition to these patient-focused activities, we also organized community meetings periodically to improve awareness of TB and available services under the project.

For peer review only - http://bmjopen.bmj.com/site/about/guidelines.xhtml

The project team was led by a program manager, who supervised three project managers responsible for operations, community engagement and monitoring and evaluation. Project manager for operations managed a team of block coordinators (BCs), one for each block in the intervention region, who managed a team of 6-7 field coordinators (FCs). Each FC covered a population of around 45,000, was responsible for training and supervision of 40-50 ASHAs and also helped with patient monitoring and community mobilization. In addition, the team included three data entry operators (DEO), data coordinator (DC), lab technician and a sputum carrier.[Figure 3]

data

Patient data. We recorded individual patient information related to referral, screening, diagnosis, and treatment follow-up in paper forms. These were linked by a unique patient identifier and maintained in separate patient folders along with copies of patient's diagnostic records. Each FC maintained folders for patients in their respective catchment areas, which were audited weekly by the BC. Trained DEOs entered data from completed forms in a patient database designed in Microsoft Excel 2016. Two DEOs checked at least one-fifth of records entered in the database for completeness and errors introduced during data entry. In addition, the DC also conducted monthly audits of the patient database.

Cost data. Each expense was first recorded on a paper-based voucher. A project manager verified each voucher, assigned it to one of the budget categories—staffing, activities (e.g., training programs), health commodities and services (GeneXpert, CXR), and administrative overheads—and entered the information in a computer-based accounting software, Tally 11®. The program manager reconciled monthly expenses against project budget.

Program data. We obtained data on quarterly TB case notifications for each block from the district program office. We also extracted monthly data on three maternal and child health indicators representing ASHA's key activities from NHM Health Statistics Information Portal.[24] These included number of pregnant women registered for antenatal care (ANC), number of institutional deliveries, and number of immunization sessions where ASHA was present.

For peer review only - http://bmjopen.bmj.com/site/about/guidelines.xhtml

analysis

We calculated the quarterly flow of patients at each stage of the care pathway: referrals eligible for screening, patients screened, presumptive TB patients identified, patients tested, patients with confirmed TB diagnosis, and confirmed TB cases initiated on treatment. We defined the pre-diagnostic loss to follow-up as the proportion of presumptive TB patients who were not tested, and the pre-treatment loss to follow-up as the proportion of patients diagnosed with TB who were not initiated on treatment. We used the number of notified TB cases to calculate annual case notification rates per 100,000 population for IR and CR.

We calculated the quarterly averages for indicators on ASHAs' performance and mapped them to baseline period (Q3 of 2016 to Q2 of 2017) and study period (Q3 of 2017 to Q2 of 2018).

To calculate the intervention cost, we included all components of operational expenses (i.e., excluding capital expenditure) that were incurred over and above routine programmatic activities under RNTCP. We divided these costs between case-finding and treatment categories based either on actuals or on the amount of time spent by the staff on the different activities estimated through semi-structured interviews.[Supplementary file 1]

We divided FCs' workday into three components: travel, case-finding activities, and treatment support activities. We estimated the time spent on the latter two based on actual time taken for each activity per patient and average patient load per FC. We calculated travel time based on the average monthly travel reimbursement amount and allocated to it between case-finding and treatment support activities in proportion to their time spent on each of these. Similar analysis was repeated for BCs and project managers with some salient differences. We did not consider travel expenses for BCs and project managers as the amount of time spent by them on travel was minimal. The time spent by these staff members in supervision was allocated to case-finding and treatment support activities in proportion of the time allocated by their team members on these two categories. Finally, the data management's time was divided into case-finding and treatment support categories in proportion to the total time spent by FCs, BCs, and project managers.[Supplementary file 2]

patient and public involvement

We neither involved patients in study design nor in the interpretation of findings.

For peer review only - http://bmjopen.bmj.com/site/about/guidelines.xhtml

FINDINGS

From July 2017 to June 2018, the project received 12393 referrals eligible for screening. Of these, 11222 patients were screened for symptoms of TB, 9884 patients with symptoms of TB were identified. Of these, 5852 patients were tested for TB whereas the remaining 40.7% were classified as pre-diagnostic loss to follow up. Of those tested, 1224 patients were diagnosed with TB with 51.2% of those being confirmed with a microbiological test. Of the diagnosed patients, 1198 patients were initiated on TB treatment yielding a pre-treatment loss to follow-up of only 2.1%.[Figure 4]

Notification rate in IR increased from 45.8 at baseline to 105.8 during study period per 100000 population but decreased from 50.7 to 45.3 in CR. Similarly, the annual notification rate per 100,000 population based on microbiological confirmation increased from 20.4 to 40.2 in IR but decreased from 29.3 to 22.8 in CR.[Table 2]

Table 2

TB case notification rates per 100,000 population in the public sector in the intervention and control region of the active case-finding project

Year	Quarter	IR		CR	
		Bac+	All cases	Bac+	All cases
2016	Q3	5.8	11.8	7.5	13.9
	Q4	4.3	9.8	5.7	11.7
2017	Q1	5.4	11.4	7.6	12.4
	Q2	4.9	12.8	8.5	12.7
	Q3	7.2	22.3	6.1	10.2
	Q4	9.5	26	5.4	9.5
2018	Q1	9.6	27.7	6	12.6
	Q2	13.9	29.8	5.3	13

IR: Intervention region

CR: Control region

Bac+: Microbiologically-confirmed TB cases

For peer review only - http://bmjopen.bmj.com/site/about/guidelines.xhtml

The overall average cost per diagnosed patient over the duration of the project was USD135.4, varying from a minimum of USD114.1 in Q3 2017 to a maximum of USD155.3 in Q4 2017. The main contributors of the cost were human resources (37.4%) and medical commodities (32.5%). Project activities and administrative overhead contributed to 20.1% and 10% of the cost, respectively.[Table 3]

For peer review only

Table 3

Costs incurred in the active case-finding program from Q3 of 2017 to Q2 of 2018.

Categories	2017 Q3	2017 Q4	2018 Q1	2018 Q2	Total	Proportion
Activities	INR 4,36,337	INR 5,04,376	INR 6,23,593	INR 6,72,826	INR 22,37,132	20.1%
Administrative overheads	INR 3,34,235	INR 2,77,192	INR 2,53,173	INR 2,42,278	INR 11,06,878	10.0%
Human resources	INR 10,53,515	INR 11,67,181	INR 9,34,772	INR 9,96,832	INR 41,52,300	37.4%
Commodities (drugs and diagnostics)	INR 3,46,683	INR 11,83,689	INR 13,05,790	INR 7,70,858	INR 36,07,020	32.5%
Grand Total	INR 21,70,770	INR 31,32,438	INR 31,17,328	INR 26,82,794	INR 1,11,03,330	
TB cases diagnosed	284	301	321	318	1224	
Cost per TB diagnosed (INR)	INR 7,644	INR 10,407	INR 9,711	INR 8,436	INR 9,071	
Cost per TB diagnosed (USD)	USD 114.1	USD 155.3	USD 144.9	USD 125.9	USD 135.4	

Exchange rate: 1 USD =67 INR

For peer review only

For peer review only - http://bmjopen.bmj.com/site/about/guidelines.xhtml

The number of pregnant women registered for ANC increased by 6.1% and 3.8% in IR and CR respectively. The number of institutional deliveries increased by 2.6% in IR as well as CR. Finally, the number of immunization sessions where an ASHA was present increased in IR by 0.2% but decreased by 2.8% in CR.[Table 4]

Table 4

ASHA's performance on reproductive, maternal, and child health program indicators in the intervention and control region in the active case-finding program

Indicator		Baseline	Study period	Change
Number of pregnant women registered for ANC	IR	5911	6270	6.1%
	CR	6098	6327	3.8%
Number of institutional deliveries conducted	IR	3962	4065	2.6%
	CR	3560	3654	2.6%
Number of immunisation sessions where ASHAs were present	IR	2550	2555	0.2%
	CR	2716	2639	-2.8%

All numbers are quarterly averages

Baseline period: Q3 of 2016 to Q2 of 2017

Study period: Q3 of 2017 to Q2 of 2018

IR: Intervention region

CR: Control region

ASHA: Accredited Social Health Activist

ANC: Antenatal checkup

DISCUSSION

ACF has been widely recommended for early identification and treatment of patients and several modelling studies have shown it to be cost-effective [8,14,16]. However, large-scale adoption of health interventions in resource-limited settings often requires cost-efficiency in addition to cost-effectiveness. Unfortunately, there is limited and mixed evidence on cost-efficient strategies in high prevalence, resource-limited settings [16,25]. In this paper, we report on one such intervention that leveraged existing CHWs in the health system and their knowledge about community health status to drive cost-efficiency. The intervention resulted in a significant increase in notification rate at a cost of USD135 per case diagnosed. In addition, involvement of CHW in TB services did not adversely impact their existing tasks.

For peer review only

It has been suggested that leveraging existing CHWs to integrate TB screening services with other community health programs like child immunization can be effective.[26] However, our study is one of the first to demonstrate the practical feasibility of this approach. CHWs have extensive knowledge of the health system and are also trusted members of their communities. Consequently, they can leverage their unique position by acting as patient navigators and ensuring that they complete their pathways to treatment completion.[27–29]

The unit cost of our intervention was substantially lower than that of other ACF interventions in the recent past. In Cambodia, ACF strategies using CHWs report a cost ranging from USD249 for door-to-door screening to USD316 for symptomatic.[15] A household contact investigation strategy in urban Uganda reported a cost of USD444 per additional case diagnosed.[26] One of the main drivers for the significant cost-efficiency of our intervention is that it, unlike door-to-door surveys or mass screening, relies on CHWs' experience and understanding of the community to find people at risk of TB. This approach is particularly useful and relevant in settings where TB incidence is evenly spread in the general population and it may not be possible to target specific high-risk population segments as recommended by WHO guidelines.[9] In particular, CHWs use their own social network to filter referrals from the larger population and enrich the stream of presumptive cases compared to what would have been possible with door-to-door screening. Lower loss to follow-up, mentioned earlier, also lowers the cost per case diagnosed and initiated on treatment.[30] Another Indian intervention that used CHWs to conduct door-to-door screening in a tribal population reported a cost of USD31 per patient excluding drugs and diagnostics. Similar components in our intervention costed USD91 per patient. The main driver for lower cost in that intervention was high incidence rate in the community (more than 10 times the national estimate) and a smaller catchment area (approximately 1/9th of our study population) which resulted in significantly lower staffing and administrative cost.[31,32]

In a constrained health system, there are perennial concerns about overburdening CHWs with new tasks thereby resulting in poor program outcomes on the existing tasks.[33–35] In this context, it is encouraging that involvement of CHWs in TB ACF activities did not adversely affect their performance on tasks related to maternal and child health. Our results agree with evidence from Tanzania regarding the ability of CHWs to handle multiple roles in HIV program as well as maternal and child health program. In particular, that study did not

For peer review only - http://bmjopen.bmj.com/site/about/guidelines.xhtml

find significant difference between trajectory of monthly HIV visits by CHWs after they were assigned additional tasks related to maternal and child health.[34]

Although the intervention produced encouraging results, its transition to a fully integrated component of the mainstream public health system is non-trivial and past evidence of such integration, both in India and elsewhere, is mixed.[36,37] Successful transition will require seamless interface between CHWs and senior RNCTP staff such as the STS. During the intervention, the field team enabled this link through supportive supervision of CHWs, which is known to be a major enabler for successful extension of CHWs' role to generate favorable outcomes.[38,39] Going forward, it would be crucial to develop a cadre of supervisors within the program who will fulfill this function. In the absence of this supervisory capacity, each STS will have to manage 150-200 CHWs, which may not be effective. Our analysis provides a framework of calculating the cost of building this supervisory capacity, which can be incorporated in the states' annual budgeting cycles through their project implementation plan.

The main strengths of our study emanate from the fact that our intervention was a pragmatic ACF implementation that utilized existing CHWs in the health system. The study was conducted in a routine programmatic site, which simulated a typical low-resource setting environment with a regular health system. We also utilized routine programmatic data on case notifications for impact evaluation and also on other health outputs to capture any externality on provision of other health services. We used a comparable control region within the same district to obtain the incremental effect of the intervention over and above other secular changes in program implementation. Finally, we had access to granular activity-level costing data, which limited (but did not eliminate) the need to allocate indirect costs.

However, our study also has some limitations. First, it was not designed as a randomized control trial. We purposively chose blocks in the IR based on the catchment area of the prior work done by the community-based organization that led this intervention. The CR, though similar to the IR in many important and relevant aspects, was also purposively chosen. As a result, we cannot rigorously claim that the impact calculated from our study is caused by the intervention and is representative at the state or national level. Second, we focused only on the incremental health system cost incurred by the intervention and did not include patient costs incurred or averted as well as costs incurred by the RNTCP to coordinate with our intervention. Finally, limited duration of our intervention did not allow us to capture longer-

For peer review only - http://bmjopen.bmj.com/site/about/guidelines.xhtml

term health outcomes such as successful treatment completion and reduced incidence, which have been documented in previous studies involving CHWs.[40–42] Careful accounting of these costs and benefits would be needed to conduct a comprehensive cost-effectiveness analysis of a national scale-up of our intervention from a societal perspective.

CONCLUSION

Existing CHWs in the health system can be leveraged to detect additional TB cases through active case finding in a cost-efficient manner. Appropriate and supportive supervision can ensure that the intervention does not adversely affect the delivery of other healthcare services in their portfolio. National scale-up of such intervention should budget for additional supervisory staff to ensure integration of CHWs' work with the senior staff in the national TB program.

For peer review only

For peer review only - http://bmjopen.bmj.com/site/about/guidelines.xhtml

ADDITIONAL INFORMATION

acknowledgement

We thank the RNTCP, Government of Bihar, State TB Officer Dr. (Major) K. N. Sahai, Communicable Disease Officer Dr. Sree Ram Prasad, and Dr. Miranda Brouwer for their support. We also acknowledge the efforts of the ASHAs and the project team at Innovators In Health. This work was presented at the 50th Union World Conference on Lung Health 2019 and we thank the audience for their comments.

competing interest

None declared

funding

This project was supported by the Stop TB Partnership's TB REACH initiative and was funded by the Government of Canada and the Bill & Melinda Gates Foundation. Manish Bhardwaj is supported by a Grand Challenges Explorations grant number OPP1190735 from the Bill & Melinda Gates Foundation.

ethics approval

A separate ethics approval was not obtained as we used aggregate intervention and programmatic data for our analysis. The project was approved by the appropriate government officials.

author contribution

Conceptualization of the intervention: TG, MB

Conceptualization of the analysis: TG, SD

Data collection: TG

Data analysis and interpretation: TG, SD

Writing — original draft: TG

Writing — review and edits: TG, SD, MB

data sharing

The cost data used in the study is published in Supplementary File 1. The aggregate indictors used to evaluate program are available from the corresponding author on reasonable request.

For peer review only

REFERENCES

1 World Health Organization. *Global tuberculosis report 2019*. World Health Organization 2019. https://apps.who.int/iris/handle/10665/329368 (accessed 18 Oct 2019).

2 World Health Organization. *Implementing the end TB strategy: the essentials*. Geneva: : World Health Organization 2015. https://apps.who.int/iris/handle/10665/206499

3 Houben RMGJ, Menzies NA, Sumner T, *et al.* Feasibility of achieving the 2025 WHO global tuberculosis targets in South Africa, China, and India: a combined analysis of 11 mathematical models. *The Lancet Global Health* 2016;**4**:e806–15. doi:10/f9b2vh

4 Yuen CM, Amanullah F, Dharmadhikari A, *et al.* Turning off the tap: stopping tuberculosis transmission through active case-finding and prompt effective treatment. *The Lancet* 2015;**386**:2334–43. doi:10/f73mmd

5 Wells WA. Onions and prevalence surveys: how to analyze and quantify tuberculosis case-finding gaps. *The International Journal of Tuberculosis and Lung Disease* 2017;**21**:1101–13. doi:10.5588/ijtld.17.0271

6 Reid MJA, Arinaminpathy N, Bloom A, *et al.* Building a tuberculosis-free world: The Lancet Commission on tuberculosis. *The Lancet* 2019;**393**:1331–84. doi:10/gfxc87

7 Reid MJ, Shah NS. Approaches to tuberculosis screening and diagnosis in people with HIV in resource-limited settings. *The Lancet Infectious Diseases* 2009;**9**:173–84. doi:10/fhkc6t

8 Ho J, Fox GJ, Marais BJ. Passive case finding for tuberculosis is not enough. *International Journal of Mycobacteriology* 2016;**5**:374–8. doi:10/gcpfsf

9 World Health Organization. Systematic screening for active tuberculosis: principles and recommendations. 2013.

10 Golub JE, Mohan CI, Comstock GW, *et al.* Active case finding of tuberculosis: historical perspective and future prospects. *The International Journal of Tuberculosis and Lung Diseas* 2005;**9**:1183–203.

11 Uplekar M, Creswell J, Ottmani S-E, *et al.* Programmatic approaches to screening for active tuberculosis [State of the art series. Active case finding/screening. Number 6 in the series]. *The International Journal of Tuberculosis and Lung Disease* 2013;**17**:1248–56. doi:10/f5hbxr

12 Saunders MJ, Tovar MA, Collier D, *et al.* Active and passive case-finding in tuberculosis-affected households in Peru: a 10-year prospective cohort study. *The Lancet Infectious Diseases* 2019;**19**:519–28. doi:10/gf6mj8

13 Kranzer K, Lawn SD, Meyer-Rath G, *et al.* Feasibility, Yield, and Cost of Active Tuberculosis Case Finding Linked to a Mobile HIV Service in Cape Town, South Africa: A Cross-sectional Study. *PLoS Medicine* 2012;**9**:e1001281. doi:10/gdxs8c

14 Azman AS, Golub JE, Dowdy DW. How much is tuberculosis screening worth? Estimating the value of active case finding for tuberculosis in South Africa, China, and India. *BMC Medicine* 2014;:9. doi:10/gb33tt

For peer review only

15 James R, Khim K, Boudarene L, *et al.* Tuberculosis active case finding in Cambodia: a pragmatic, cost-effectiveness comparison of three implementation models. *BMC Infect Dis* 2017;**17**:580. doi:10/gdxs8g

16 Dobler CC. Screening strategies for active tuberculosis: focus on cost-effectiveness. *ClinicoEconomics and Outcomes Research* 2016;**8**:335–47. doi:10/gdxtmw

17 Registrar General of India. Census of India 2011. Office of the Registrar General Census Commissioner, India 2011.

18 Socio-Economic Caste Census 2011. Ministry of Rural Development, Government of India https://secc.gov.in/ (accessed 31 Aug 2019).

19 Annual Health Survey 2011-12, Bihar Factsheet. New Delhi: : Office of the Registrar General & Census Commissioner http://www.censusindia.gov.in/vital_statistics/AHSBulletins/AHS_Factsheets_2011_12/Bihar_Factsheet_2011-12.pdf (accessed 3 Feb 2019).

20 International Institute for Population Sciences. NFHS 4 Factsheet Samastipur. International Institute for Population Sciences http://rchiips.org/nfhs/FCTS/BR/BR_FactSheet_221_Samastipur.pdf (accessed 3 Feb 2019).

21 Central TB Division. India TB Report 2018. New Delhi, India: : Directorate General of Health Services, Ministry of Health and Family Welfare, Government of India 2018. https://tbcindia.gov.in/showfile.php?lid=3314 (accessed 18 Nov 2019).

22 Blok L, Creswell J, Stevens R, *et al.* A pragmatic approach to measuring, monitoring and evaluating interventions for improved tuberculosis case detection. *International Health* 2014;**6**:181–8. doi:10.1093/inthealth/ihu055

23 District TB Report, Samastipur. District TB Cell, Revised National TB Control Program 2017.

24 Ministry of Health & Family Welfare, Government of India. NHM Health Statistics Information Portal. NHM Health Statistics Information Portal. https://nrhm-mis.nic.in/SitePages/Home.aspx (accessed 4 Feb 2019).

25 Lung T, Marks GB, Nhung NV, *et al.* Household contact investigation for the detection of tuberculosis in Vietnam: economic evaluation of a cluster-randomised trial. *The Lancet Global Health* 2019;**7**:e376–84. doi:10/gf5tdz

26 Sekandi JN, Dobbin K, Oloya J, *et al.* Cost-effectiveness analysis of community active case finding and household contact investigation for tuberculosis case detection in urban Africa. *PLoS ONE* 2015;**10**:e0117009. doi:10.1371/journal.pone.0117009

27 Mishra A. 'Trust and teamwork matter': Community health workers' experiences in integrated service delivery in India. *Global Public Health* 2014;**9**:960–74. doi:10/gf6k98

28 Yellapa V, Devadasan N, Krumeich A, *et al.* How patients navigate the diagnostic ecosystem in a fragmented health system: a qualitative study from India. *Global Health Action* 2017;**10**:1350452. doi:10/gf2pcd

For peer review only - http://bmjopen.bmj.com/site/about/guidelines.xhtml

29 Subbaraman R, Nathavitharana RR, Mayer KH, *et al.* Constructing care cascades for active tuberculosis: A strategy for program monitoring and identifying gaps in quality of care. *PLOS Medicine* 2019;**16**:e1002754. doi:10/gf38sz

30 Subbaraman R, Nathavitharana RR, Satyanarayana S, *et al.* The Tuberculosis Cascade of Care in India's Public Sector: A Systematic Review and Meta-analysis. *PLOS Medicine* 2016;**13**:e1002149. doi:10/f9b29f

31 Vyas A, Creswell J, Codlin AJ, *et al.* Community-based active case-finding to reach the most vulnerable: tuberculosis in tribal areas of India. *The International Journal of Tuberculosis and Lung Disease* 2019;**23**:750–755. doi:10.5588/ijtld.18.0741

32 Sohn H, Vyas A, Puri L, *et al.* Costs and operation management of community outreach program for tuberculosis in tribal populations in India. *Public Health Action* 2019;**9**:58–62. doi:10/gf5zst

33 Shelley KD, Mpembeni R, Frumence G, *et al.* Integrating Community Health Worker Roles to Improve Facility Delivery Utilization in Tanzania: Evidence from an Interrupted Time Series Analysis. *Maternal and Child Health Journal* 2019;**23**:1327–38. doi:10/gf5xfh

34 Shelley KD, Frumence G, Mpembeni R, *et al.* Can volunteer community health workers manage multiple roles? An interrupted time-series analysis of combined HIV and maternal and child health promotion in Iringa, Tanzania. *Health Policy and Planning* 2018;**33**:1096–106. doi:10/gf5xd7

35 Singh S, Dwivedi N, Dongre A, *et al.* Functioning and time utilisation by female multi-purpose health workers in South India: a time and motion study. *Human Resources for Health* 2018;**16**:64. doi:10/gf6mbt

36 Scott K, George AS, Ved RR. Taking stock of 10 years of published research on the ASHA programme: examining India's national community health worker programme from a health systems perspective. *Health Research Policy and Systems* 2019;**17**:29. doi:10/gf2t58

37 Sundaram N, James R, Sreynimol U, *et al.* A strong TB programme embedded in a developing primary healthcare system is a lose-lose situation: insights from patient and community perspectives in Cambodia. *Health Policy Plan* 2017;**32**:ii32–42. doi:10.1093/heapol/czx079

38 Scott K, Beckham SW, Gross M, *et al.* What do we know about community-based health worker programs? A systematic review of existing reviews on community health workers. *Human Resources for Health* 2018;**16**:39. doi:10.1186/s12960-018-0304-x

39 Adejumo AO, Azuogu B, Okorie O, *et al.* Community referral for presumptive TB in Nigeria: a comparison of four models of active case finding. *BMC Public Health* 2016;**16**:177. doi:10.1186/s12889-016-2769-7

40 Datiko DG, Lindtjørn B. Health Extension Workers Improve Tuberculosis Case Detection and Treatment Success in Southern Ethiopia: A Community Randomized Trial. *PLOS One* 2009;**4**:e5443. doi:10/d9zb95

For peer review only - http://bmjopen.bmj.com/site/about/guidelines.xhtml

41 Musa BM, Iliyasu Z, Yusuf SM, *et al.* Systematic review and metanalysis on community based interventions in tuberculosis care in developing countries. *Niger J Med* 2014;**23**:103–17.

42 Alipanah N, Jarlsberg L, Miller C, *et al.* Adherence interventions and outcomes of tuberculosis treatment: A systematic review and meta-analysis of trials and observational studies. *PLOS Medicine* 2018;**15**:e1002595. doi:10/gdsx3v

For peer review only

For peer review only - http://bmjopen.bmj.com/site/about/guidelines.xhtml

LEGEND FOR FIGURES

figure 1

Map indicating the blocks in intervention and control region in Samastipur district, Bihar.

figure 2

The diagnostic protocol used in the active case-finding project.

Legend: T/T: Treatment; F/U: Follow-up; EPTB: Extrapulmonary TB; DSTB: Drug-sensitive TB; DRTB: Drug-resistant TB; PLHIV: People living with HIV; CXR(?): Irrespective of the CXR result

figure 3

The organization chart in the active case-finding project

figure 4

The patient care cascade from Q3 2017 to Q2 2018

* All percentages are calculated as a proportion of the number of participants entering the previous step of the cascade

For peer review only

For peer review only - http://bmjopen.bmj.com/site/about/guidelines.xhtml

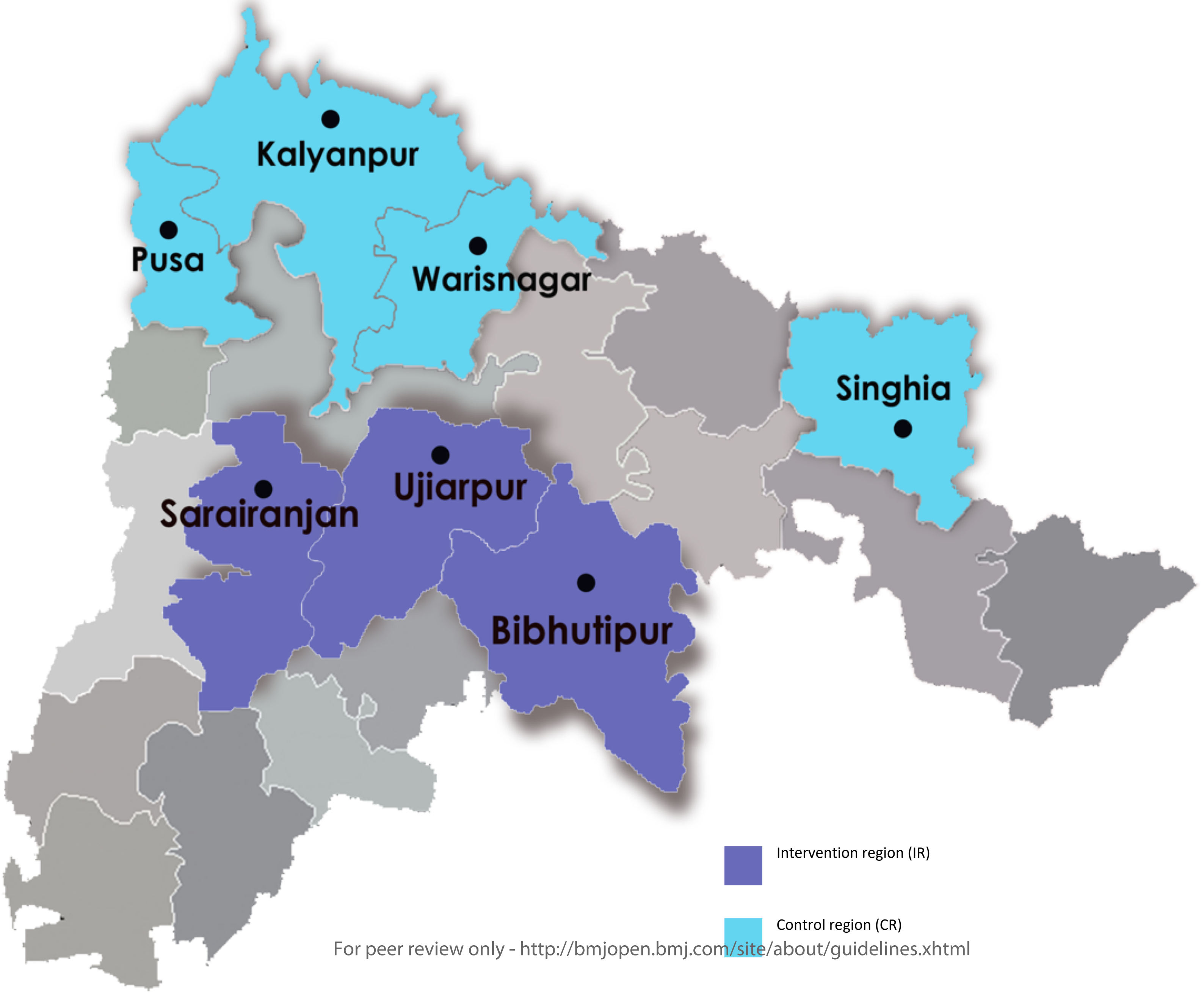

For peer review only - http://bmjopen.bmj.com/site/about/guidelines.xhtml

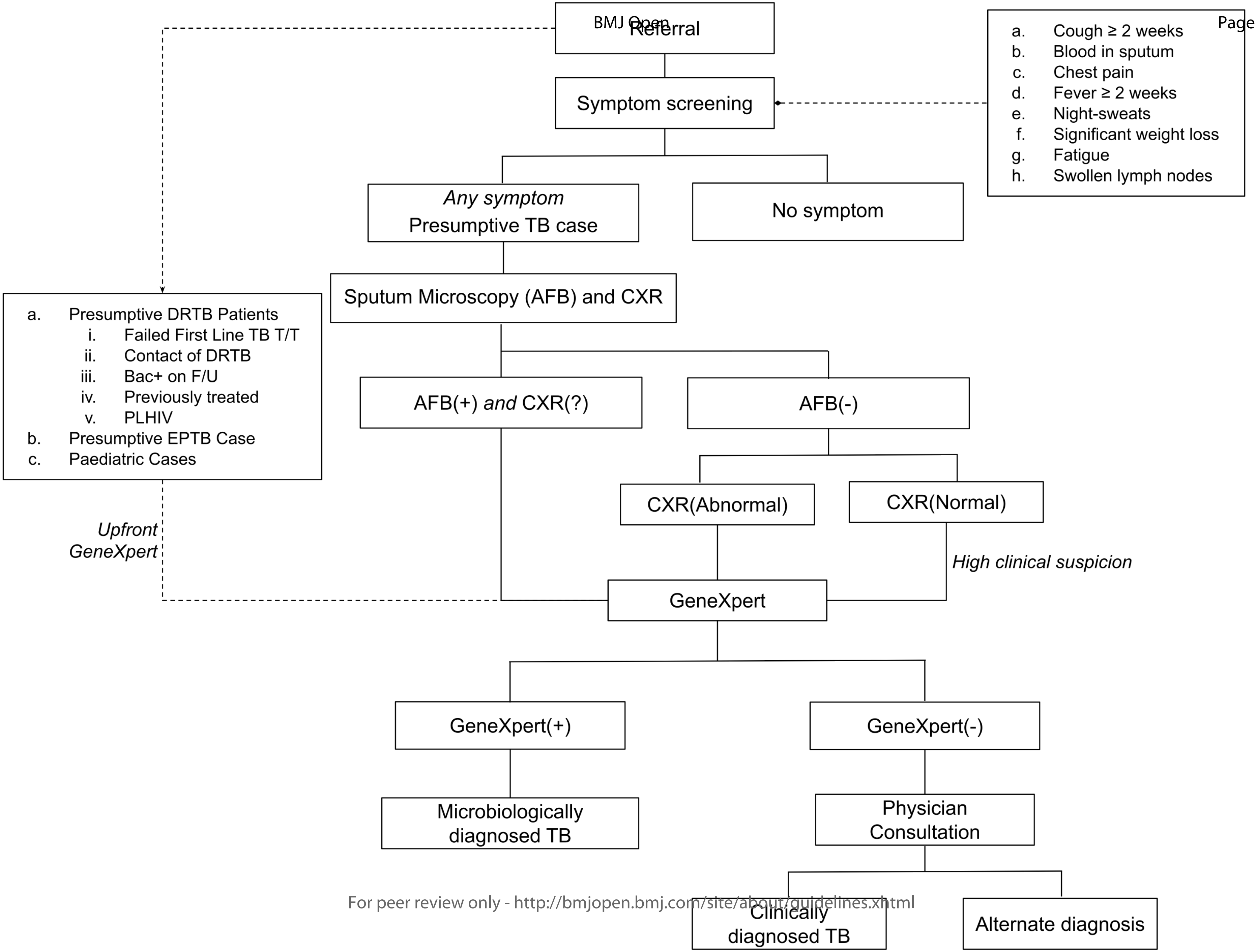
Referral
Symptom screening
a. Cough ≥ 2 weeks
b. Blood in sputum
c. Chest pain
d. Fever ≥ 2 weeks
e. Night-sweats
f. Significant weight loss
g. Fatigue
h. Swollen lymph nodes
Any symptom
Presumptive TB case
No symptom
Sputum Microscopy (AFB) and CXR
a. Presumptive DRTB Patients
i. Failed First Line TB T/T
ii. Contact of DRTB
iii. Bac+ on F/U
iv. Previously treated
v. PLHIV
b. Presumptive EPTB Case
c. Paediatric Cases
AFB(+) and CXR(?)
AFB(-)
CXR(Abnormal)
CXR(Normal)
Upfront GeneXpert
High clinical suspicion
GeneXpert
GeneXpert(+)
GeneXpert(-)
Microbiologically diagnosed TB
Physician Consultation
Clinically diagnosed TB
Alternate diagnosis

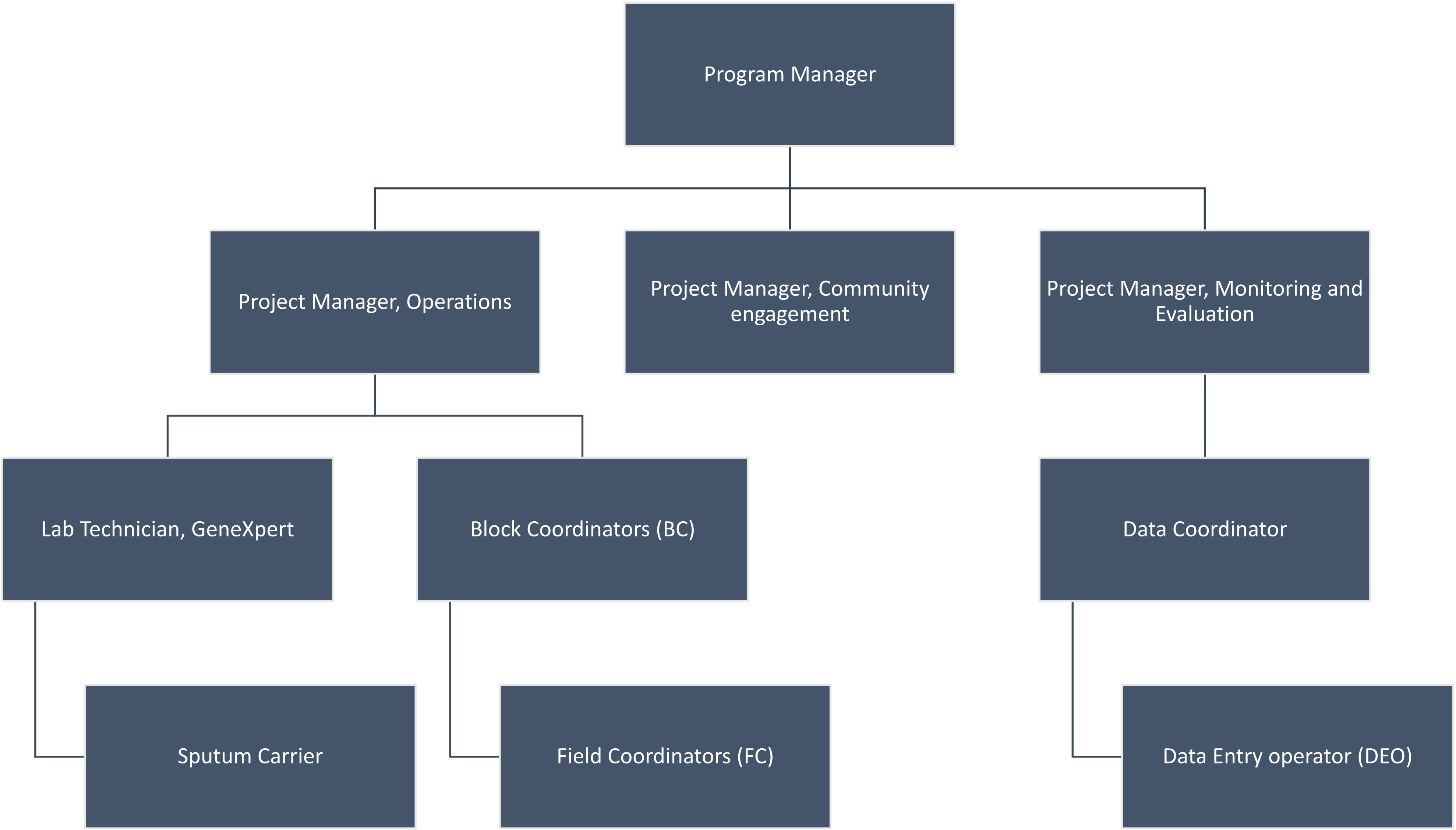
Program Manager
Project Manager, Operations
Project Manager, Community engagement
Project Manager, Monitoring and Evaluation
Lab Technician, GeneXpert
Block Coordinators (BC)
Data Coordinator
Sputum Carrier
Field Coordinators (FC)
Data Entry operator (DEO)

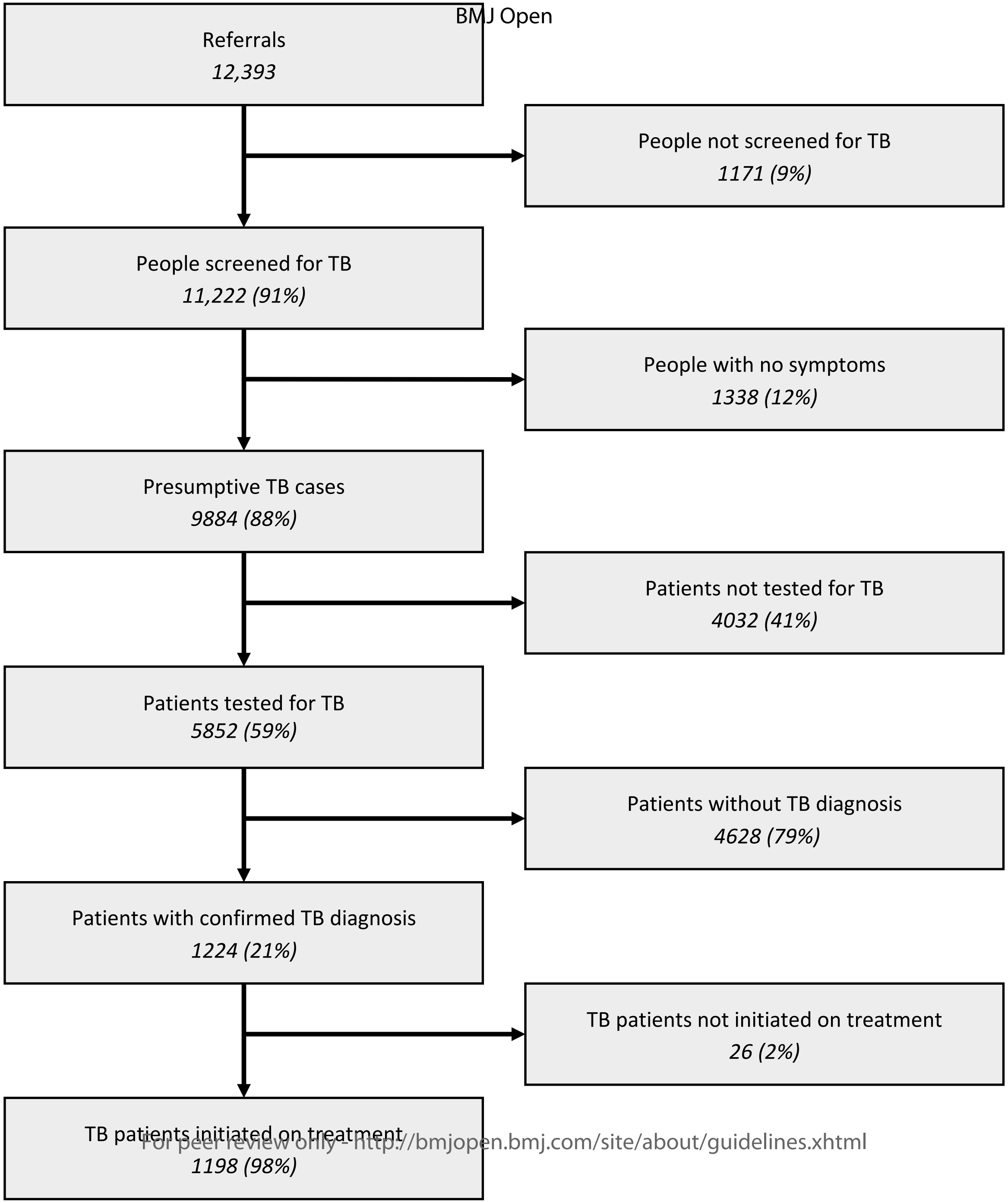

For peer review only - http://bmjopen.bmj.com/site/about/guidelines.xhtml

Supplementary File 1: The cost breakdown of the active case-finding program from Q3 2017 to Q2 2018 with cost allocation between case-finding and treatment support activities

									Cost allocation			Case-finding costs proportionate to the cost allocation				
#	Category	Item	2017Q3	2017Q4	2018Q1	2018Q2	Total	Comment	Case-finding	Treatment	Blank	C2017Q3	C2017Q4	C2018Q1	C2018Q2	Ctotal
1	Activities	Trainers, per-diem costs	₹ 29,850	₹ 29,850	₹ 29,850	₹ 29,850	₹ 1,19,400		100%	0%		₹ 29,850	₹ 29,850	₹ 29,850	₹ 29,850	₹ 1,19,400
2	Activities	Case-finding incentives	₹ 1,42,000	₹ 1,50,500	₹ 1,60,500	₹ 1,59,000	₹ 6,12,000	Unit cost INR500	100%	0%		₹ 1,42,000	₹ 1,50,500	₹ 1,60,500	₹ 1,59,000	₹ 6,12,000
3	Activities	Treatment completion incentives	₹ 2,65,050	₹ 2,82,150	₹ 2,98,300	₹ 2,92,600	₹ 11,38,100	Unit cost INR1000	0%	100%		₹ -	₹ -	₹ -	₹ -	₹ -
4	Activities	Field visits (fuel costs, management)	₹ 2,71,541	₹ 3,11,828	₹ 3,69,122	₹ 3,23,806	₹ 12,76,297		66%	34%		₹ 1,80,029	₹ 2,06,739	₹ 2,44,724	₹ 2,14,680	₹ 8,46,171
5	Activities	Training and workshops	₹ 65,395	₹ 52,488	₹ 58,690	₹ 2,69,343	₹ 4,45,916		55%	45%		₹ 35,925	₹ 28,835	₹ 32,242	₹ 1,47,965	₹ 2,44,967
6	Activities	Transport allowance	₹ 24,059	₹ 73,053	₹ 1,06,012	₹ 1,04,381	₹ 3,07,505		100%	0%		₹ 24,059	₹ 73,053	₹ 1,06,012	₹ 1,04,381	₹ 3,07,505
7	Activities	Communication material	₹ 24,474	₹ 15,400	₹ 50,265	₹ 16,950	₹ 1,07,089		100%	0%		₹ 24,474	₹ 15,400	₹ 50,265	₹ 16,950	₹ 1,07,089
8	Administrative overheads	Information technology (mobile data, voice)	₹ 25,020	₹ 25,638	₹ 16,969	₹ 13,150	₹ 80,777		66%	34%		₹ 16,588	₹ 16,998	₹ 11,250	₹ 8,718	₹ 53,554
9	Administrative overheads	Car rental (per month)	₹ 1,50,000	₹ 1,50,000	₹ 1,50,000	₹ 1,50,000	₹ 6,00,000		81%	19%		₹ 1,21,835	₹ 1,21,835	₹ 1,21,835	₹ 1,21,835	₹ 4,87,339
10	Administrative overheads	Rent	₹ 43,900	₹ 51,900	₹ 51,900	₹ 51,900	₹ 1,99,600		81%	19%		₹ 35,657	₹ 42,155	₹ 42,155	₹ 42,155	₹ 1,62,121
11	Administrative overheads	Electricity	₹ 4,085	₹ 8,055	₹ 7,740	₹ 7,654	₹ 27,534		81%	19%		₹ 3,318	₹ 6,543	₹ 6,287	₹ 6,217	₹ 22,364
12	Administrative overheads	Supplies (stationery, workshops, etc.)	₹ 1,93,094	₹ 1,10,390	₹ 88,210	₹ 78,000	₹ 4,69,694		81%	19%		₹ 1,56,837	₹ 89,662	₹ 71,647	₹ 63,354	₹ 3,81,500
13	Human resources	Program manager	₹ 2,10,000	₹ 2,10,000	₹ 2,10,000	₹ 2,10,000	₹ 8,40,000		81%	19%		₹ 1,70,569	₹ 1,70,569	₹ 1,70,569	₹ 1,70,569	₹ 6,82,275
14	Human resources	Project manager (community and training)	₹ 1,35,000	₹ 1,35,000	₹ 1,35,000	₹ 2,03,226	₹ 6,08,226		81%	19%		₹ 1,09,651	₹ 1,09,651	₹ 1,09,651	₹ 1,65,067	₹ 4,94,020
15	Human resources	Project manager (service delivery)	₹ 2,10,484	₹ 2,70,000	₹ 1,46,613	₹ 1,35,000	₹ 7,62,097		81%	19%		₹ 1,70,962	₹ 2,19,303	₹ 1,19,084	₹ 1,09,651	₹ 6,18,999
16	Human resources	GeneXpert technician	₹ 56,903	₹ 1,02,000	₹ 1,02,000	₹ 1,02,000	₹ 3,62,903		100%	0%		₹ 56,903	₹ 1,02,000	₹ 1,02,000	₹ 1,02,000	₹ 3,62,903
17	Human resources	MIS operator	₹ 49,484	₹ 90,250	₹ 1,01,000	₹ 1,16,250	₹ 3,56,984		66%	34%		₹ 32,807	₹ 59,835	₹ 66,962	₹ 77,073	₹ 2,36,677
18	Human resources	Consultants	₹ 1,66,833	₹ 1,65,000	₹ -	₹ -	₹ 3,31,833		100%	0%		₹ 1,66,833	₹ 1,65,000	₹ -	₹ -	₹ 3,31,833
19	Human resources	Block coordinators (BCs)	₹ 1,92,000	₹ 1,92,000	₹ 1,92,000	₹ 1,92,000	₹ 7,68,000		62%	38%		₹ 1,19,897	₹ 1,19,897	₹ 1,19,897	₹ 1,19,897	₹ 4,79,588
20	Human resources	Field coordinators (FCs)	₹ 4,11,196	₹ 4,02,155	₹ 4,48,906	₹ 4,59,767	₹ 17,22,024		55%	45%		₹ 2,25,893	₹ 2,20,926	₹ 2,46,609	₹ 2,52,576	₹ 9,46,005
21	Procurement of medical items	GeneXpert, test cartridge	₹ 1,29,006	₹ 6,26,923	₹ 6,20,888	₹ 3,39,489	₹ 17,16,306	Unit cost USD11.26	100%	0%		₹ 1,29,006	₹ 6,26,923	₹ 6,20,888	₹ 3,39,489	₹ 17,16,306
22	Procurement of medical items	Contingency, drugs	₹ 38,127	₹ 98,864	₹ 1,28,695	₹ 1,65,859	₹ 4,31,545		0%	100%		₹ -	₹ -	₹ -	₹ -	₹ -
23	Procurement of medical items	Contingency, chest X-rays	₹ 1,32,823	₹ 2,57,945	₹ 3,39,280	₹ 2,11,545	₹ 9,41,593		100%	0%		₹ 1,32,823	₹ 2,57,945	₹ 3,39,280	₹ 2,11,545	₹ 9,41,593
24	Procurement of medical items	Contingency, sputum microscopy	₹ 11,280	₹ 3,980	₹ 13,400	₹ 9,200	₹ 37,860		100%	0%		₹ 11,280	₹ 3,980	₹ 13,400	₹ 9,200	₹ 37,860
25	Procurement of medical items	Extra-pulmonary TB diagnostics	₹ 10,986	₹ 33,907	₹ 73,800	₹ 69,324	₹ 1,88,017		100%	0%		₹ 10,986	₹ 33,907	₹ 73,800	₹ 69,324	₹ 1,88,017
26	Procurement of medical items	Complications and hospitalization	₹ 25,719	₹ 45,597	₹ 42,386	₹ 36,111	₹ 1,49,813		0%	100%		₹ -	₹ -	₹ -	₹ -	₹ -
27	Procurement of medical items	Sputum containers (for transport)	₹ 8,894	₹ -	₹ -	₹ -	₹ 8,894		100%	0%		₹ 8,894	₹ -	₹ -	₹ -	₹ 8,894
28	Procurement of medical items	Customs duty and Xpert shipping	₹ 53,694	₹ 2,60,934	₹ 2,58,422	₹ 1,41,300	₹ 7,14,350	Unit cost INR314	100%	0%		₹ 53,694	₹ 2,60,934	₹ 2,58,422	₹ 1,41,300	₹ 7,14,350
29		*Additional information*														
30		Cartridges used	171	831	823	450										
31		Total TB diagnosed	284	301	321	318										
32		Total TB treatments started	279	297	314	308		See Figure 4								
33		Total treatment completed	265	282	298	293		Assumed at 95%								

* All cost figures are reported in Indian Rupee (INR or ₹)
* The cost allocation is sourced from the time calculations in Supplementary File 2
* The case-finding incentive (item 2) is derived from total cases diagnosed (item 31)
* The treatment completion incentive (item 3) is derived from total treatment completed (item 33)
* The GeneXpert costs (item 21 and 28) are derived from the actual consumption of the cartridges (item 30)

For peer review only

Supplementary File 2: The time allocation of human resource between case-finding and treatment support activities

Table A: Detailed time allocation for human resource

#	Human Resource	Activities	Value	Comment
1	**Field coordinator (FC)**	Work hours (in hours)	48	6 working days, 8 hours a day; 5 day field work + 1 day reporting-review-planning-training
		Total patients diagnosed per month	105	100 - 110 patients diagnosed per month
		Average treatment duration per patient (in months)	7	DSTB treatment duration of 6 - 8 months
		Patients per field coordinator, steady state	35	18 field coordinators in the program
		Travel per week (in hours)	193	4 weeks in a month. Average travel reimbursement per field coordinator of ₹2700 at ₹3.5 per km.
1.1		**Travel time per week (in hours)**	10	Average speed of 20 kmph
1.2		**Treatment support activities**		
		Time per visit (in hours)	1	Half hour on average
		Number of visits per patient in a month	3	3 times a month
		Total time spent per week in treatment support activities (in hours)	13	
1.3		**Case-finding activities**		
		Time spent in ASHA (CHW) training per week (in hours)	2	Average 1 meeting per week lasting nearly 2 hour
		Time spent in meeting individual ASHAs per week (in hours)	2	4 ASHAs per week, 0.5 hour per meeting
		Time spent in hospital visits and assisting in diagnosis per week (in hours)	6	3 times a week, 2 hours per visit
		Time spent in meeting referrals and screening per week (in hours)	6	12 per week, 0.5 hour per visit
		Total time spent per week in case-finding activities (in hours)	16	
1.4		**Reporting-review-planning-training, time spent per week (in hours)**	8	1 day
2	**Block coordinator (BC)**	Work hours (in hours)	48	6 working days, 8 hours a day
2.1		Admin-reporting-review-planning, time spent per week (in hours)	8	1 complete day, focused around FCs in their area
2.2		Block review-data management-project meeting-training, time spent per week (in hours)	8	Project meeting once a week, data management with MIS team, reviewing project performance, training
2.3		Supervisory work, time spent per week (in hours)	32	Split in proportion to FCs time spent in activities
3	**Managers**	Work hours	48	6 working days, 8 hours a day
3.1		Review-planning-design, time spent per week (in hours)	24	3 days a week, largely centered around case-finding
3.2		Supervisory work, time spent per week (in hours)	24	Split in proportion to BCs time spent in activities
4	**Data management team**	Work hours	48	Split in proportion of FCs time under reporting-review-planning

For peer review only

Table B: Summary of time allocation between case-finding and treatment support activities

Field coordinator (FC)	Activity	Time	Travel	Reporting-review-planning-training	Total	Proportion	Assumptions
1	Case-finding activities	16.0	5.3	4.4	25.7	55%	* Note
2	Treatment support activities	13.1	4.3	3.6	21.1	45%	

Block coordinator (BC)	Activity	Admin-reporting-review-planning	Block review-data management-project meeting-training	Supervisory work, time spent per week	Proportion
1	Case-finding activities	4.4	8.0	17.6	62%
2	Treatment support activities	3.6	0.0	14.4	38%

Managers	Activity	Review-planning-design	Supervisory work	Proportion
1	Case-finding activities	24	15.0	81%
2	Treatment support activities	0	9.0	19%

Data management team	Activity	Time	Proportion
1	Case-finding activities	94.7	66%
2	Treatment support activities	48.1	34%

Table C: Proportion of time allocation for human resource

#	Human Resource	Case-finding	Treatment support
1	Field coordinator (FC)	55%	45%
2	Block coordinator (BC)	62%	38%
3	Managers	81%	19%
4	Data management team	66%	34%

* **Note**: Time spent in travel and reporting-review-planning-training is proportionate to time spent in respective case-finding and treatment activities. It should be noted that FCs would have likely travelled to a village for both activities.

For peer review only

BMJ Open

Role of community health workers in improving cost efficiency in an active case finding tuberculosis program: An operational research study from rural Bihar, India

Journal:	*BMJ Open*
Manuscript ID	bmjopen-2019-036625.R1
Article Type:	Original research
Date Submitted by the Author:	24-Jun-2020
Complete List of Authors:	Garg, Tushar; Innovators In Health, Research<br>Bhardwaj, Manish; Innovators In Health, Operations<br>Deo, Sarang; Indian School of Business, Operations Management
**Primary Subject Heading**:	Public health
Secondary Subject Heading:	Health economics, Global health, Infectious diseases
Keywords:	Public health < INFECTIOUS DISEASES, Tuberculosis < INFECTIOUS DISEASES, PUBLIC HEALTH, Health economics < HEALTH SERVICES ADMINISTRATION & MANAGEMENT, International health services < HEALTH SERVICES ADMINISTRATION & MANAGEMENT, Organisation of health services < HEALTH SERVICES ADMINISTRATION & MANAGEMENT

SCHOLARONE™ Manuscripts

I, the Submitting Author has the right to grant and does grant on behalf of all authors of the Work (as defined in the below author licence), an exclusive licence and/or a non-exclusive licence for contributions from authors who are: i) UK Crown employees; ii) where BMJ has agreed a CC-BY licence shall apply, and/or iii) in accordance with the terms applicable for US Federal Government officers or employees acting as part of their official duties; on a worldwide, perpetual, irrevocable, royalty-free basis to BMJ Publishing Group Ltd ("BMJ") its licensees and where the relevant Journal is co-owned by BMJ to the co-owners of the Journal, to publish the Work in this journal and any other BMJ products and to exploit all rights, as set out in our licence.

The Submitting Author accepts and understands that any supply made under these terms is made by BMJ to the Submitting Author unless you are acting as an employee on behalf of your employer or a postgraduate student of an affiliated institution which is paying any applicable article publishing charge ("APC") for Open Access articles. Where the Submitting Author wishes to make the Work available on an Open Access basis (and intends to pay the relevant APC), the terms of reuse of such Open Access shall be governed by a Creative Commons licence – details of these licences and which Creative Commons licence will apply to this Work are set out in our licence referred to above.

Other than as permitted in any relevant BMJ Author's Self Archiving Policies, I confirm this Work has not been accepted for publication elsewhere, is not being considered for publication elsewhere and does not duplicate material already published. I confirm all authors consent to publication of this Work and authorise the granting of this licence.

For peer review only

TITLE

Role of community health workers in improving cost efficiency in an active case finding tuberculosis program: An operational research study from rural Bihar, India

MANUSCRIPT TYPE

Research article

AUTHORS

Tushar Garg[1], Manish Bhardwaj[1], Sarang Deo[2]

[1] Innovators In Health, Patna

[2] Indian School of Business, Hyderabad

ADDRESS FOR CORRESPONDENCE

Tushar Garg,

Innovators in Health, #176, Ground Floor, Road #2,

New Pataliputra Colony, Patna – 800013. Bihar. India.

Phone: +91 98286 09562

Email: tgarg@innovatorsinhealth.org

MANUSCRIPT DETAILS

Abstract: 291 words

Manuscript: 3752 words

Tables: 4

Figures: 4

KEYWORDS

Accredited Social Health Activist, ASHA, Community Health Workers, Tuberculosis Active Case Finding, Cost Analysis

For peer review only

ABSTRACT

Objectives

Cost-efficient active case finding (ACF) approaches are needed for their large-scale adoption in national tuberculosis (TB) programs. Our aim was to assess if community health workers' (CHW) knowledge about families' health status can improve the cost-efficiency of the ACF program without adversely affecting the delivery of other health services for which they are responsible.

Design

Quasi-experimental design

Interventions

We evaluated an ACF program in the Samastipur district in Bihar, India, between July 2017 and June 2018. CHWs called Accredited Social Health Activists generated referrals of individuals at risk of TB and conducted symptom-based screening to identify presumptive TB patients. They also helped them undergo testing and provided treatment support for confirmed TB cases.

Primary and secondary outcome measures

We compared the notification rate from the intervention region with that from a control region in the same district with similar characteristics. We analyzed operational data to calculate the cost per TB case diagnosed. We used routine programmatic data from the public health system to estimate the impact on other services provided by CHWs.

Findings

CHWs identified 9895 presumptive TB patients. Of these, 5864 patients were tested for TB, and 1236 were confirmed as TB cases. Annual public case notification rate increased sharply in the intervention region from 45.8 to 105.8 per 100,000 population, whereas it decreased from 50.7 to 45.3 in the control region. There was no practically or statistically significant impact on other output indicators of the CHWs, such as institutional deliveries (-0.04%). The overall cost of the intervention was about USD134 per diagnosed case. Main cost drivers were human resources, and commodities (drugs and diagnostics), which contributed 37.4% and 32.5% of the cost, respectively.

Conclusions

ACF programs that utilize existing CHWs in the health system are feasible, cost-efficient, and do not adversely affect other healthcare services delivered by CHWs.

For peer review only

STRENGTHS AND LIMITATIONS OF THIS STUDY

1. A pragmatic active case-finding implementation that utilized existing community health workers in the health system.
2. Used a comparable control region to obtain the incremental effect of the intervention.
3. Purposively selected areas, hence, not a randomized control trial.
4. Patient costs incurred or averted and the national tuberculosis program costs not included.
5. The small scale of the study and geographical location limit generalizability

For peer review only

For peer review only - http://bmjopen.bmj.com/site/about/guidelines.xhtml

INTRODUCTION

The World Health Organization (WHO) estimates about 10 million people were falling ill with tuberculosis (TB) and nearly 1.5 million dying of it in 2018.[1] Despite the continuous increase in case notifications in recent years, the 2018 estimates predict a gap of as much as 30% between the incident and notified cases globally. Progress toward WHO's target of a 90% reduction in TB incidence rate by 2035 is severely limited by existing passive case-finding (PCF) approaches that wait for patients to seek care at a health facility.[2–4] As a result, these approaches fail to address significant barriers in accessing care, such as poor geographical and financial access, stigma, and poor awareness.[5]

Active case-finding (ACF) can address these challenges by finding previously undetected cases and promptly initiating treatment.[4,6,7] Modeling studies estimate that such strategies can decrease TB incidence.[8,9] In contrast with passive approaches, ACF is a health system initiated screening process that uses context-specific diagnostic algorithms and accommodates various implementation strategies, including mass radiography, contact investigation, and house-to-house surveys.[6,10,11] Although modelling studies have shown ACF strategies to be cost-effective, cost per diagnosed case of such programs can be very high, thereby limiting their large-scale adoption.[8,12–14] As a result, there is limited empirical evidence from high-burden and resource-constrained settings to inform key operational decisions regarding ACF programs: who will conduct ACF activities, how will they be integrated within the health system, and how will these additional activities impact other health services.[15].

In this study, we address these questions with evidence from a novel intervention in rural India that leveraged existing Community Health Workers (CHWs) in the public health system for ACF activities. In particular, our aim is to assess if CHWs' knowledge about health statuses of families can improve the cost-efficiency of the ACF program without adversely affecting the delivery of other health services for which they are responsible.

METHODS

Study design

Our intervention was implemented from May 15th, 2017, with state and district health administration's approval as an extension of routine services provided as part of the Revised National TB Control Program (RNTCP). We used a quasi-experimental design to evaluate the impact of the intervention over a period between July 1st, 2017 and June 30th, 2018. We

For peer review only - http://bmjopen.bmj.com/site/about/guidelines.xhtml

utilized the period between May 15th, 2017 and July 1st, 2017 in preparatory activities to launch the intervention.

Study setting

Our study was conducted in the Samastipur district of the East Indian state of Bihar. In 2011, it had a population of about 42.6 million, of which 96.5% lived in rural areas. The literacy rate was 50.3%, and the sex ratio was 911.[16] The main source of income in more than 71.3% households was casual labor, and the highest individual income was less than INR5000 (USD71) in 69.1% of households.[17] The total fertility rate in the district was 3.8, and the infant mortality rate was 53 deaths per 1000 live births.[18] More than 70% of births occurred at a healthcare institution.[19] In 2017, the annual TB case notification rate for the district was 55 per 100,000 population with a pre-treatment loss to follow-up (PTLFU) rate of 25%. In 2016, a successful treatment outcome was reported for 72% of the microbiologically confirmed (Bac+) new TB cases (44% of all cases).[20]

The intervention region (IR) consisted of three blocks—Ujiarpur, Bibhutipur, and Sarairanjan—with a total population of 1,021,483.[Figure 1]. We chose four blocks—Kalyanpur, Warisnagar, Pusa, and Singhia—as the control region (CR) with a population of 981,924.[21] The choice of these blocks was purposive with an emphasis on a similar population, sociodemographic and health system characteristics, and TB epidemiology. These were geographically separated from the IR to minimize the spill-over effects of the intervention.

IR and CR were similar along relevant sociodemographic variables such as the proportion of the population belonging to scheduled castes (18.2% vs. 20.8%).[17][Table 1] Further, the structure of the public health systems in IR and CR was comparable on relevant dimensions. Each block in IR, as well as CR, coincided with a Tuberculosis Unit (TU) under the Revised National TB Control Program (RNTCP), which was managed by a Senior Treatment Supervisor (STS). IR and CR included four designated microscopy centers (DMCs) each, where sputum microscopy was provided. Finally, the annual TB case notification rate was comparable across IR and CR (52 vs. 53.1 per 100,000 population in 2016).[22]

For peer review only - http://bmjopen.bmj.com/site/about/guidelines.xhtml

Table 1

The demographic characteristics of the intervention and control region in the active case-finding project

Characteristics	Intervention region	Control region
Blocks	3	4
Area (sq. km.)	582	623
Population	1021483	981924
Sex ratio	918	919
Proportion of scheduled castes population	18.2%	20.8%
Literacy rate	63.5%	59.8%
Households with monthly income of highest earning household member less than INR5000	69.8%	70.6%

Intervention

We implemented an ACF intervention with the support of RNTCP and the National Health Mission (NHM) and project funding from Stop TB Partnership's TB REACH. Under this intervention, we engaged with community health workers (CHWs), locally known as Accredited Social Health Activists (ASHAs), who work for the NHM. Their main role was community mobilization and facilitating last-mile delivery of health services across multiple programs though their focus is reproductive, maternal, and child health. Although ASHAs were chosen from literate women between 25 and 45 years of age with a preference to those educated up to the tenth standard, the criteria were relaxed if no such woman was available in the village.[23] They received performance- and activity-linked remuneration, for example, USD0.7 to report a newborn death within 24 hours, UDS2 to attend review meetings, USD8 for antenatal care and institutional delivery, up to USD15 for promoting contraception, and up to USD75 supporting TB treatment (USD15 for a new case, USD22 for a previously-diagnosed case, and USD75 for a drug-resistant TB case).[24,25] They were supervised by ASHA facilitators—one each for about 20 ASHAs—and a block community mobilizer at the block level.

We trained these ASHAs to identify patients with TB symptoms during their routine work and refer them to a field coordinator (FC). The FCs further screened these patients using a symptom-based tool after obtaining their verbal consent.[10] Presumptive TB patients

For peer review only - http://bmjopen.bmj.com/site/about/guidelines.xhtml

identified through screening were accompanied by ASHAs to the nearest PHC for diagnostic testing and physician consultation. All presumptive TB patients underwent sputum microscopy and chest X-ray (CXR). GeneXpert testing, if indicated by the diagnostic protocol, was conducted at the laboratory operated using project resources.[Figure 2] Even if CXR and sputum microscopy results were not abnormal, physicians could order a GeneXpert based on the clinical presentation. We used the standard diagnostic algorithm that is recommended by the RNTCP.[26] However, RNTCP recommendation of universal drug susceptibility testing by GeneXpert for all TB cases was being rolled out in phases and was only available in the IR as a part of the intervention.[27]

Upon confirmation of TB diagnosis, ASHA obtained drugs from the STS and initiated treatment at patient's residence. For each confirmed case of TB, the project paid INR200 (USD3) to ASHA for referral and INR300 (USD4.5) to ASHA for assisting in diagnosis and treatment initiation. ASHAs counselled patients on the importance of adherence and treatment completion and monitored them for adverse effects through regular follow-up household visits. They received INR400 (USD6) after their first follow-up visit and INR600 (USD9) upon successful completion of treatment. In addition to these patient-focused activities, we also organized community meetings periodically to improve awareness of TB and available services under the project.

The project team was led by a program manager, who supervised three project managers responsible for operations, community engagement and monitoring and evaluation. Project manager for operations managed a team of block coordinators (BCs), one for each block in the intervention region, who managed a team of 6-7 field coordinators (FCs). Each FC covered a population of around 50,000, was responsible for training and supervision of 35-45 ASHAs, and helped with patient monitoring and community mobilization. Supervision involved visiting patients along with ASHA, assisting the ASHAs in keeping record and filing RNTCP paperwork, and assisting ASHAs in troubleshooting across the care pathway. Also, the team included three data entry operators (DEO), data coordinator (DC), lab technician, and a sputum carrier.[Figure 3]

Cost framework

We used a top-down approach from the provider perspective for costing that included only costs incurred in the intervention. We defined cost-efficiency in operational terms of cost per

For peer review only - http://bmjopen.bmj.com/site/about/guidelines.xhtml

case detected to distinguish it from the more conventional term of cost-effectiveness, which is typically measured as cost per QALY (quality-adjusted life years) gained or DALY (disability-adjusted life years) averted.

Data

Patient data. We recorded individual patient information related to referral, screening, diagnosis, and treatment follow-up in paper forms. These were linked by a unique patient identifier and maintained in separate patient folders along with copies of the patient's diagnostic records. Each FC maintained folders for patients in their respective catchment areas, which were audited weekly by the BC. Trained DEOs entered data from completed forms in a patient database designed in Microsoft Excel 2016. Two DEOs checked at least one-fifth of records entered in the database for completeness and errors introduced during data entry. Besides, DC also conducted monthly audits of the patient database. Appropriate measures were taken to ensure safe-keeping of the confidential patient records.

Cost data. Each expense was first recorded on a paper-based voucher. A project manager verified each voucher, assigned it to one of the budget categories—staffing, activities (e.g., training programs), health commodities and services (GeneXpert, CXR), and administrative overheads—and entered the information in computer-based accounting software, Tally 11®. The program manager reconciled monthly expenses against the project budget.

Program data. We obtained data on quarterly TB case notifications for each block from the district program office. We also extracted monthly data on three maternal and child health indicators representing ASHA's key activities from the NHM Health Statistics Information Portal.[28] These included the number of pregnant women registered for antenatal care (ANC), the number of institutional deliveries, and the number of immunization sessions where ASHA was present. The program data was obtained after the intervention, whereas the patient and cost data were collected in tandem with the intervention.

Analysis

We calculated the quarterly flow of patients at each stage of the care pathway: referrals eligible for screening, patients screened, presumptive TB patients identified, patients tested, patients with confirmed TB diagnosis, and confirmed TB cases initiated on treatment. We defined the pre-diagnostic loss to follow-up as the proportion of presumptive TB patients

For peer review only - http://bmjopen.bmj.com/site/about/guidelines.xhtml

who were not tested, and the pre-treatment loss to follow-up as the proportion of patients diagnosed with TB who were not initiated on treatment. We used the number of notified TB cases to calculate annual case notification rates per 100,000 population for IR and CR.

We calculated the quarterly averages for indicators on ASHAs' performance and mapped them to the baseline period (Q3 of 2016 to Q2 of 2017) and the study period (Q3 of 2017 to Q2 of 2018).

To calculate the intervention cost, we included all components of operational expenses (i.e., excluding capital expenditure) that were incurred over and above routine programmatic activities under RNTCP. We divided these costs between case-finding and treatment categories based on actuals or the amount of time spent by the staff on the different activities estimated through semi-structured interviews. We used an exchange rate of INR67 per USD for all costs.[Supplementary file 1]

We divided FCs' workday into three components: travel, case-finding activities, and treatment support activities. We estimated the time spent on the latter two based on actual time taken for each activity per patient and average patient load per FC. We calculated travel time based on the average monthly travel reimbursement amount and allocated to it between case-finding and treatment support activities in proportion to their time spent on each of these. A similar analysis was repeated for BCs and project managers with some salient differences. We did not consider travel expenses for BCs and project managers as the amount of time spent by them on travel was minimal. The time spent by these staff members in supervision was allocated to case-finding and treatment support activities in the proportion of the time allocated by their team members on these two categories. Finally, the data management's time was divided into case-finding and treatment support categories in proportion to the total time spent by FCs, BCs, and project managers.[Supplementary file 2]

Patient and public involvement

We neither involved patients in study design nor in the interpretation of findings.

FINDINGS

From July 2017 to June 2018, the project received 12394 referrals eligible for screening. Of these, 11233 patients were screened for symptoms of TB, 9895 patients with symptoms of TB were identified. Of these, 5864 patients were tested for TB, whereas the remaining

For peer review only - http://bmjopen.bmj.com/site/about/guidelines.xhtml

40.7% were classified as the pre-diagnostic loss to follow up. Of those tested, 1236 patients were diagnosed with TB, with 51.5% of those being confirmed with a microbiological test. Of the diagnosed patients, 1194 patients were initiated on TB treatment yielding a pre-treatment loss to follow-up of only 3.4%.[Figure 4][Supplementary file 3, 4]

The notification rate in IR increased from 45.8 at baseline to 105.8 during the study period per 100000 population but decreased from 50.7 to 45.3 in CR. Similarly, the annual notification rate per 100,000 population for microbiologically-confirmed TB increased from 20.4 to 40.2 in IR but decreased from 29.3 to 22.8 in CR.[Table 2]

Table 2

TB case notification rates per 100,000 population in the public sector in the intervention and control region of the active case-finding project

Year	Quarter	IR		CR	
		Bac+	All cases	Bac+	All cases
2016	Q3	5.8	11.8	7.5	13.9
	Q4	4.3	9.8	5.7	11.7
2017	Q1	5.4	11.4	7.6	12.4
	Q2	4.9	12.8	8.5	12.7
	Q3	7.2	22.3	6.1	10.2
	Q4	9.5	26	5.4	9.5
2018	Q1	9.6	27.7	6	12.6
	Q2	13.9	29.8	5.3	13

IR: Intervention region

CR: Control region

Bac+: Microbiologically-confirmed TB cases

The overall average cost per diagnosed patient over the duration of the project was USD133.9, varying from a minimum of USD114 in Q3 2017 to a maximum of USD154.7 in Q4 2017. The main contributors to the cost were human resources (37.4%) and medical commodities (32.5%). Project activities and administrative overhead contributed to 20% and 10% of the cost, respectively.[Table 3]

For peer review only - http://bmjopen.bmj.com/site/about/guidelines.xhtml

Table 3

Costs incurred in the active case-finding program from Q3 of 2017 to Q2 of 2018.

Categories	2017 Q3	2017 Q4	2018 Q1	2018 Q2	Total	Proportion
Activities	□4,33,837	□5,01,876	□6,20,093	□6,66,326	□22,22,132	20.0%
Administrative overheads	□3,34,235	□2,77,192	□2,53,173	□2,42,279	□11,06,879	10.0%
Human resources	□10,53,515	□11,67,181	□9,34,772	□9,96,832	□41,52,300	37.4%
Commodities (drugs and diagnostics)	□3,46,683	□11,83,689	□13,05,790	□7,70,858	□36,07,020	32.5%
Grand Total	□21,68,270	□31,29,938	□ 31,13,828	□26,76,295	□1,10,88,331	
TB cases diagnosed	284	302	324	326	1236	
Cost per TB diagnosed (INR)	□7,635	□10,364	□9,611	□8,209	□8,971	
Cost per TB diagnosed (USD)	$114.0	$154.7	$143.4	$122.5	$133.9	

Exchange rate: 1 USD ($) = 67 INR (□)

For peer review only

For peer review only - http://bmjopen.bmj.com/site/about/guidelines.xhtml

The number of pregnant women registered for ANC increased by 6.1% and 3.8% in IR and CR, respectively. The number of institutional deliveries increased by 2.6% in IR as well as CR. Finally, the number of immunization sessions where an ASHA was present increased in IR by 0.2% but decreased by 2.8% in CR.[Table 4]

Table 4

ASHA's performance on reproductive, maternal, and child health program indicators in the intervention and control region in the active case-finding program

Indicator		Baseline	Study period	Change
Number of pregnant women registered for ANC	IR	5911	6270	6.1%
	CR	6098	6327	3.8%
Number of institutional deliveries conducted	IR	3962	4065	2.6%
	CR	3560	3654	2.6%
Number of immunisation sessions where ASHAs were present	IR	2550	2555	0.2%
	CR	2716	2639	-2.8%

All numbers are quarterly averages

Baseline period: Q3 of 2016 to Q2 of 2017

Study period: Q3 of 2017 to Q2 of 2018

IR: Intervention region

CR: Control region

ASHA: Accredited Social Health Activist

ANC: Antenatal checkup

DISCUSSION

ACF has been widely recommended for the early identification and treatment of patients.[29] Several modelling studies in various contexts, including India, China, and Uganda, have shown it to be cost-effective.[8,15,30,31] However, large-scale adoption of health interventions in resource-limited settings often requires cost-efficiency in addition to cost-effectiveness. Unfortunately, there is limited and mixed evidence on cost-efficient strategies in high prevalence, resource-limited settings [15,32]. In this paper, we report on one such intervention that leveraged existing CHWs in the health system and their knowledge about community health status to drive cost-efficiency. The intervention resulted in a significant

For peer review only - http://bmjopen.bmj.com/site/about/guidelines.xhtml

increase in the notification rate at the cost of about USD134 per case diagnosed. In addition, the involvement of CHW in TB services did not adversely impact their existing tasks.

It has been suggested that leveraging existing CHWs to integrate TB screening services with other community health programs like child immunization can be effective.[31] However, our study is one of the first to demonstrate the practical feasibility of this approach. CHWs have extensive knowledge of the health system and are also trusted members of their communities. Consequently, they can leverage their unique position by acting as patient navigators and ensuring that they complete their pathways to treatment completion.[33–35] Involving CHWs can also aid in engaging other actors like informal health providers and community in the way they referred people to be screened in our intervention. Their role, although, was ancillary while the FCs screened and diagnosis and treatment activities for such cases were undertaken by the CHWs.

The unit cost of our intervention was substantially lower than that of other ACF interventions in the recent past. In Cambodia, ACF strategies using CHWs report a cost ranging from USD249 for door-to-door screening to USD316 for symptomatic.[14] A household contact investigation strategy in urban Uganda reported a cost of USD444 per additional case diagnosed.[31] One of the main drivers for the significant cost-efficiency of our intervention is that it, unlike door-to-door surveys or mass screening, relies on CHWs' experience and understanding of the community to find people at risk of TB. This approach is particularly useful and relevant in settings where TB incidence is evenly spread in the general population, and it may not be possible to target specific high-risk population segments as recommended by WHO guidelines.[10] In particular, CHWs use their own social network to filter referrals from the larger population and enrich the stream of presumptive cases compared to what would have been possible with door-to-door screening. The lower loss to follow-up, mentioned earlier, also lowers the cost per case diagnosed and initiated on treatment.[36] Another Indian intervention that used CHWs to conduct door-to-door screening in a tribal population reported a cost of USD31 per patient, excluding drugs and diagnostics. Similar components in our intervention costed USD91 per patient. The main driver for lower cost in that intervention was a high incidence rate in the community (more than ten times the national estimate) and a smaller catchment area (approximately 1/9th of our study population), which resulted in significantly lower staffing and administrative cost.[37,38] However, the difference in costs needs to be interpreted with caution as studies vary substantially in their context (choice of ACF strategy, intervention design and diagnostic

For peer review only - http://bmjopen.bmj.com/site/about/guidelines.xhtml

algorithm, TB epidemiology, and health system characteristics) as well as their costing methodology (costing perspective (patient, provider, societal) and outcome measure).[39,40]

The key factors in explaining the efficacy of ASHAs in case finding in our intervention are the incentives and the level of supervision that they received. The amount of incentive to ASHAs was competitive in comparison to their other activities, as shown above. However, its untimely disbursal is a shortcoming of the routine programs and likely a reason ASHA partnered with our intervention, where such disbursals were prompt.[41] Further, ASHA's motivation is also dependent on her social and contextual environment, amongst other factors, and it was also experienced in our implementation.[42,43] ASHA's engagement level varied widely and it likely impacted the yield as well. Nonetheless, her remuneration is not considered commensurate with those of other health personnel and, overall, insufficient for the work they put in.[44–46] The drivers of engagement and the role of incentives in the poorly understood decision-making process of ASHA deserves further investigation. In a constrained health system, there are perennial concerns about overburdening CHWs with new tasks, thereby resulting in poor program outcomes on the existing tasks.[47–49] In this context, it is encouraging that involvement of CHWs in TB ACF activities did not adversely affect their performance on tasks related to maternal and child health. Any changes in indicators were small and of limited pragmatic significance.[Table 4] Our results agree with evidence from Tanzania regarding the ability of CHWs to handle multiple roles in the HIV program as well as maternal and child health programs. In particular, that study did not find a significant difference between the trajectory of monthly HIV visits by CHWs after they were assigned additional tasks related to maternal and child health.[48] However, any integration of CHWs in other programs should carefully assess factors affecting their capacity and performance. In India, their training and education levels vary widely, and poor motivation and inadequate supportive supervision are well known limiting factors.[50–52]

Although the intervention produced encouraging results, there was heterogeneity in the performance metrics across the blocks, over time, and across ASHAs.[Supplementary file 4] Further efforts are needed to understand this heterogeneity better and use it for benchmarking and program improvement. Moreover, addressing the pre-diagnostic loss to follow-up will likely improve the yield in such a program. Its responsible factors are poor support at the family and health centre level, inadequate services in the health system, and stigma.[53] Future efforts should focus on empowerment of ASHAs and patients, and

For peer review only - http://bmjopen.bmj.com/site/about/guidelines.xhtml

ameliorating the health system deficiencies. Its transition to a fully integrated component of the mainstream public health system is non-trivial, and past evidence of such integration, both in India and elsewhere, is mixed.[54,55] A successful transition will a require seamless interface between CHWs and senior RNCTP staff, such as the STS. During the intervention, the field team enabled this link through supportive supervision of CHWs, which is known to be a major enabler for the successful extension of CHWs' role to generate favorable outcomes.[56,57] Going forward, it would be crucial to develop a cadre of supervisors within the program who will fulfill this function. In the absence of this supervisory capacity, each STS will have to manage 150-200 CHWs, which may not be effective. Our analysis provides a framework for calculating the cost of building this supervisory capacity, which can be incorporated in the states' annual budgeting cycles through their project implementation plan.

The main strengths of our study emanate from the fact that our intervention was a pragmatic ACF implementation that utilized existing CHWs in the health system. The study was conducted in a routine programmatic site, which simulated a typical low-resource setting environment with a regular health system. We also utilized routine programmatic data on case notifications for impact evaluation and other health outputs to capture any externality on the provision of other health services. We used a comparable control region within the same district to obtain the intervention's incremental effect over and above other secular changes in program implementation. Finally, we had access to granular activity-level costing data, which limited (but did not eliminate) the need to allocate indirect costs.

However, our study also has some limitations. First, it was not designed as a randomized control trial. We purposively chose blocks in the IR based on the catchment area of the prior work done by the community-based organization that led this intervention. The CR, though similar to the IR in many important and relevant aspects, was also purposively chosen. As a result, we cannot rigorously claim that the impact calculated from our study is caused by the intervention and is representative at the state or national level. Second, we focused only on the incremental health system cost incurred by the intervention and did not include patient costs incurred or averted as well as costs incurred by the RNTCP to coordinate with our intervention. Finally, the limited duration of our intervention did not allow us to capture longer-term health outcomes such as successful treatment completion and impact on TB epidemiology. Careful accounting of these costs and benefits would be needed to conduct a

For peer review only - http://bmjopen.bmj.com/site/about/guidelines.xhtml

comprehensive cost-effectiveness analysis of a national scale-up of our intervention from a societal perspective.

CONCLUSION

Leveraging existing CHWs in the health system can enhance cost-efficiency of tuberculosis active case finding programs without adversely affecting the delivery of other healthcare services in their portfolio. National scale-up of this approach for tuberculosis active case finding will require detailed understanding of existing capacity utilization of CHWs due to their routine tasks and the importance of supportive supervision in helping them effectively manage the new task in addition to the routine ones.

For peer review only

ADDITIONAL INFORMATION

Acknowledgement

We acknowledge the efforts of the Accredited Health Social Activists and the project team at Innovators In Health. This work was presented at the 50th Union World Conference on Lung Health 2019, and we thank the audience for their comments.

Competing interest

None declared

Funding

This project was supported by the Stop TB Partnership's TB REACH initiative and was funded by the Government of Canada and the Bill & Melinda Gates Foundation. Manish Bhardwaj was supported by a Grand Challenges Explorations grant number OPP1190735 from the Bill & Melinda Gates Foundation. Neither funders had any role in the study design, data collection and analysis, decision to publish, or preparation of the manuscript.

Ethics approval

Ethical approval was obtained from the Institutional Review Board at Indian School of Business, Hyderabad. The board waived the informed consent requirement for the study. Further, only aggregate intervention data was used for the analysis.

Author contribution

Funding acquisition: MB, TG
Conceptualization of the intervention: TG, MB
Conceptualization of the analysis: TG, SD
Data collection: TG
Data analysis and interpretation: TG, SD
Writing — original draft: TG
Writing — review and edits: TG, SD, MB

Data sharing

The cost, program, and yield data used in the study are available in Supplementary Files 1, 3, 4, respectively.

For peer review only

REFERENCES

1 World Health Organization. *Global tuberculosis report 2019*. World Health Organization 2019. https://apps.who.int/iris/handle/10665/329368 (accessed 18 Oct 2019).

2 World Health Organization. *Implementing the end TB strategy: the essentials*. Geneva: : World Health Organization 2015. https://apps.who.int/iris/handle/10665/206499

3 Houben RMGJ, Menzies NA, Sumner T, *et al.* Feasibility of achieving the 2025 WHO global tuberculosis targets in South Africa, China, and India: a combined analysis of 11 mathematical models. *The Lancet Global Health* 2016;**4**:e806–15. doi:10/f9b2vh

4 Yuen CM, Amanullah F, Dharmadhikari A, *et al.* Turning off the tap: stopping tuberculosis transmission through active case-finding and prompt effective treatment. *The Lancet* 2015;**386**:2334–43. doi:10/f73mmd

5 Wells WA. Onions and prevalence surveys: how to analyze and quantify tuberculosis case-finding gaps. *The International Journal of Tuberculosis and Lung Disease* 2017;**21**:1101–13. doi:10.5588/ijtld.17.0271

6 Uplekar M, Creswell J, Ottmani S-E, *et al.* Programmatic approaches to screening for active tuberculosis [State of the art series. Active case finding/screening. Number 6 in the series]. *The International Journal of Tuberculosis and Lung Disease* 2013;**17**:1248–56. doi:10/f5hbxr

7 Kranzer K, Afnan-Holmes H, Tomlin K, *et al.* The benefits to communities and individuals of screening for active tuberculosis disease: a systematic review. *Int J Tuberc Lung Dis* 2013;**17**:432–46. doi:10.5588/ijtld.12.0743

8 Azman AS, Golub JE, Dowdy DW. How much is tuberculosis screening worth? Estimating the value of active case finding for tuberculosis in South Africa, China, and India. *BMC Medicine* 2014;:9. doi:10/gb33tt

9 Dowdy DW, Lotia I, Azman AS, *et al.* Population-Level Impact of Active Tuberculosis Case Finding in an Asian Megacity. *PLoS ONE* 2013;**8**:e77517. doi:10/gcz84s

10 World Health Organization. Systematic screening for active tuberculosis: principles and recommendations. 2013.

11 Golub JE, Mohan CI, Comstock GW, *et al.* Active case finding of tuberculosis: historical perspective and future prospects. *The International Journal of Tuberculosis and Lung Diseas* 2005;**9**:1183–203.

12 Saunders MJ, Tovar MA, Collier D, *et al.* Active and passive case-finding in tuberculosis-affected households in Peru: a 10-year prospective cohort study. *The Lancet Infectious Diseases* 2019;**19**:519–28. doi:10/gf6mj8

13 Kranzer K, Lawn SD, Meyer-Rath G, *et al.* Feasibility, Yield, and Cost of Active Tuberculosis Case Finding Linked to a Mobile HIV Service in Cape Town, South Africa: A Cross-sectional Study. *PLoS Medicine* 2012;**9**:e1001281. doi:10/gdxs8c

For peer review only - http://bmjopen.bmj.com/site/about/guidelines.xhtml

14 James R, Khim K, Boudarene L, *et al.* Tuberculosis active case finding in Cambodia: a pragmatic, cost-effectiveness comparison of three implementation models. *BMC Infect Dis* 2017;**17**:580. doi:10/gdxs8g

15 Dobler CC. Screening strategies for active tuberculosis: focus on cost-effectiveness. *ClinicoEconomics and Outcomes Research* 2016;**8**:335–47. doi:10/gdxtmw

16 Registrar General of India. Census of India 2011. Office of the Registrar General Census Commissioner, India 2011.

17 Socio-Economic Caste Census 2011. Ministry of Rural Development, Government of India https://secc.gov.in/ (accessed 31 Aug 2019).

18 Annual Health Survey 2011-12, Bihar Factsheet. New Delhi: : Office of the Registrar General & Census Commissioner http://www.censusindia.gov.in/vital_statistics/AHSBulletins/AHS_Factsheets_2011_12/Bihar_Factsheet_2011-12.pdf (accessed 3 Feb 2019).

19 International Institute for Population Sciences. NFHS 4 Factsheet Samastipur. International Institute for Population Sciences http://rchiips.org/nfhs/FCTS/BR/BR_FactSheet_221_Samastipur.pdf (accessed 3 Feb 2019).

20 Central TB Division. India TB Report 2018. New Delhi, India: : Directorate General of Health Services, Ministry of Health and Family Welfare, Government of India 2018. https://tbcindia.gov.in/showfile.php?lid=3314 (accessed 18 Nov 2019).

21 Blok L, Creswell J, Stevens R, *et al.* A pragmatic approach to measuring, monitoring and evaluating interventions for improved tuberculosis case detection. *International Health* 2014;**6**:181–8. doi:10.1093/inthealth/ihu055

22 District TB Report, Samastipur. District TB Cell, Revised National TB Control Program 2017.

23 About Accredited Social Health Activist (ASHA). National Health Mission. https://nhm.gov.in/index1.php?lang=1&level=1&sublinkid=150&lid=226 (accessed 29 May 2020).

24 Update on ASHA Programme. New Delhi: : National Health Mission, Ministy of Health and Family Welfare 2018. http://nhsrcindia.org/sites/default/files/asha%20reduced.pdf (accessed 29 Jul 2019).

25 ASHA Incentives. 2018.http://statehealthsocietybihar.org/asha/ASHAincentiveDetails.pdf (accessed 29 May 2020).

26 Revised Technical & Operational Guidelines for Tuberculosis Control in India. New Delhi: : Central TB Division 2016. https://www.tbcindia.gov.in/WriteReadData/Revised%20Technical%20and%20Operational%20Guidelines/files/assets/basic-html/page-1.html# (accessed 4 Feb 2019).

27 Guidelines on Programmatic Management of Drug-Resistant Tuberculosis in India. New Delhi: : Revised National Tuberculosis Control Programme, Central TB Division 2017.

For peer review only

For peer review only - http://bmjopen.bmj.com/site/about/guidelines.xhtml

https://tbcindia.gov.in/WriteReadData/Guideline%20for%20PMDT%20in%20India%202017.zip

28 Ministry of Health & Family Welfare, Government of India. NHM Health Statistics Information Portal. NHM Health Statistics Information Portal. https://nrhm-mis.nic.in/SitePages/Home.aspx (accessed 4 Feb 2019).

29 Reid MJA, Arinaminpathy N, Bloom A, *et al.* Building a tuberculosis-free world: The Lancet Commission on tuberculosis. *The Lancet* 2019;**393**:1331–84. doi:10/gfxc87

30 Mupere E, Schiltz NK, Mulogo E, *et al.* Effectiveness of active case-finding strategies in tuberculosis control in Kampala, Uganda. *The International Journal of Tuberculosis and Lung Disease* 2013;**17**:207–13. doi:10/gdxs8d

31 Sekandi JN, Dobbin K, Oloya J, *et al.* Cost-effectiveness analysis of community active case finding and household contact investigation for tuberculosis case detection in urban Africa. *PLoS ONE* 2015;**10**:e0117009. doi:10.1371/journal.pone.0117009

32 Lung T, Marks GB, Nhung NV, *et al.* Household contact investigation for the detection of tuberculosis in Vietnam: economic evaluation of a cluster-randomised trial. *The Lancet Global Health* 2019;**7**:e376–84. doi:10/gf5tdz

33 Mishra A. 'Trust and teamwork matter': Community health workers' experiences in integrated service delivery in India. *Global Public Health* 2014;**9**:960–74. doi:10/gf6k98

34 Yellapa V, Devadasan N, Krumeich A, *et al.* How patients navigate the diagnostic ecosystem in a fragmented health system: a qualitative study from India. *Global Health Action* 2017;**10**:1350452. doi:10/gf2pcd

35 Subbaraman R, Nathavitharana RR, Mayer KH, *et al.* Constructing care cascades for active tuberculosis: A strategy for program monitoring and identifying gaps in quality of care. *PLOS Medicine* 2019;**16**:e1002754. doi:10/gf38sz

36 Subbaraman R, Nathavitharana RR, Satyanarayana S, *et al.* The Tuberculosis Cascade of Care in India's Public Sector: A Systematic Review and Meta-analysis. *PLOS Medicine* 2016;**13**:e1002149. doi:10/f9b29f

37 Vyas A, Creswell J, Codlin AJ, *et al.* Community-based active case-finding to reach the most vulnerable: tuberculosis in tribal areas of India. *The International Journal of Tuberculosis and Lung Disease* 2019;**23**:750–755. doi:10.5588/ijtld.18.0741

38 Sohn H, Vyas A, Puri L, *et al.* Costs and operation management of community outreach program for tuberculosis in tribal populations in India. *Public Health Action* 2019;**9**:58–62. doi:10/gf5zst

39 Jo Y, Mirzoeva F, Chry M, *et al.* Standardized framework for evaluating costs of active case-finding programs: An analysis of two programs in Cambodia and Tajikistan. *PLOS ONE* 2020;**15**:e0228216. doi:10/ggxf9s

40 Cunnama L, Gomez GB, Siapka M, *et al.* A Systematic Review of Methodological Variation in Healthcare Provider Perspective Tuberculosis Costing Papers Conducted in

For peer review only

For peer review only - http://bmjopen.bmj.com/site/about/guidelines.xhtml

Low- and Middle-Income Settings, Using An Intervention-Standardised Unit Cost Typology. *PharmacoEconomics* Published Online First: 4 May 2020. doi:10/ggxj2x

41 Sarin E, Sooden A, Kole SK, *et al.* Identification of challenges and needs to improve community health workers performance: Narratives of Accredited Social Health Activists (ASHA) from two Indian districts. *Journal of Public Health in Developing Countries* 2016;**2**:173–182.

42 Abdel-All M, Angell B, Jan S, *et al.* What do community health workers want? Findings of a discrete choice experiment among Accredited Social Health Activists (ASHAs) in India. *BMJ Global Health* 2019;**4**:e001509. doi:10/gf6mbr

43 Wahid SS, Munar W, Das S, *et al.* 'Our village is dependent on us. That's why we can't leave our work'. Characterizing mechanisms of motivation to perform among Accredited Social Health Activists (ASHA) in Bihar. *Health Policy Plan* 2020;**35**:58–66. doi:10/ggxqxm

44 Bhatia K. Performance-Based Incentives of the ASHA Scheme: Stakeholders' Perspectives. *Economic and Political Weekly* 2014;**49**:145–51.

45 Sarin E, Lunsford SS, Sooden A, *et al.* The Mixed Nature of Incentives for Community Health Workers: Lessons from a Qualitative Study in Two Districts in India. *Frontiers in Public Health* 2016;**4**. doi:10/ggxqxw

46 Saprii L, Richards E, Kokho P, *et al.* Community health workers in rural India: analysing the opportunities and challenges Accredited Social Health Activists (ASHAs) face in realising their multiple roles. *Human Resources for Health* 2015;**13**:95. doi:10/f74dgf

47 Shelley KD, Mpembeni R, Frumence G, *et al.* Integrating Community Health Worker Roles to Improve Facility Delivery Utilization in Tanzania: Evidence from an Interrupted Time Series Analysis. *Maternal and Child Health Journal* 2019;**23**:1327–38. doi:10/gf5xfh

48 Shelley KD, Frumence G, Mpembeni R, *et al.* Can volunteer community health workers manage multiple roles? An interrupted time-series analysis of combined HIV and maternal and child health promotion in Iringa, Tanzania. *Health Policy and Planning* 2018;**33**:1096–106. doi:10/gf5xd7

49 Singh S, Dwivedi N, Dongre A, *et al.* Functioning and time utilisation by female multi-purpose health workers in South India: a time and motion study. *Human Resources for Health* 2018;**16**:64. doi:10/gf6mbt

50 Sarin E, Lunsford SS. How female community health workers navigate work challenges and why there are still gaps in their performance: a look at female community health workers in maternal and child health in two Indian districts through a reciprocal determinism framework. *Human Resources for Health* 2017;**15**:44. doi:10/gbpgph

51 Scott K, Shanker S. Tying their hands? Institutional obstacles to the success of the ASHA community health worker programme in rural north India. *AIDS Care* 2010;**22**:1606–12. doi:10/cg2thv

For peer review only

For peer review only - http://bmjopen.bmj.com/site/about/guidelines.xhtml

52 Sharma R, Webster P, Bhattacharyya S. Factors affecting the performance of community health workers in India: a multi-stakeholder perspective. *Global Health Action* 2014;**7**:25352. doi:10/ggxrhw

53 Garg T, Gupta V, Sen D, *et al.* Prediagnostic loss to follow-up in an active case finding tuberculosis programme: a mixed-methods study from rural Bihar, India. *BMJ Open* 2020;**10**:e033706. doi:10.1136/bmjopen-2019-033706

54 Scott K, George AS, Ved RR. Taking stock of 10 years of published research on the ASHA programme: examining India's national community health worker programme from a health systems perspective. *Health Research Policy and Systems* 2019;**17**:29. doi:10/gf2t58

55 Sundaram N, James R, Sreynimol U, *et al.* A strong TB programme embedded in a developing primary healthcare system is a lose-lose situation: insights from patient and community perspectives in Cambodia. *Health Policy Plan* 2017;**32**:ii32–42. doi:10.1093/heapol/czx079

56 Scott K, Beckham SW, Gross M, *et al.* What do we know about community-based health worker programs? A systematic review of existing reviews on community health workers. *Human Resources for Health* 2018;**16**:39. doi:10.1186/s12960-018-0304-x

57 Adejumo AO, Azuogu B, Okorie O, *et al.* Community referral for presumptive TB in Nigeria: a comparison of four models of active case finding. *BMC Public Health* 2016;**16**:177. doi:10.1186/s12889-016-2769-7

For peer review only

LEGEND FOR FIGURES

figure 1

Map indicating the blocks in intervention and control region in Samastipur district, Bihar.

figure 2

The diagnostic protocol used in the active case-finding project.

Legend: T/T: Treatment; F/U: Follow-up; EPTB: Extrapulmonary TB; DSTB: Drug-sensitive TB; DRTB: Drug-resistant TB; PLHIV: People living with HIV; CXR(?): Irrespective of the CXR result

figure 3

The organization chart in the active case-finding project

figure 4

The patient care cascade from Q3 2017 to Q2 2018

* All percentages are calculated as a proportion of the number of participants entering the previous step of the cascade

For peer review only

For peer review only - http://bmjopen.bmj.com/site/about/guidelines.xhtml

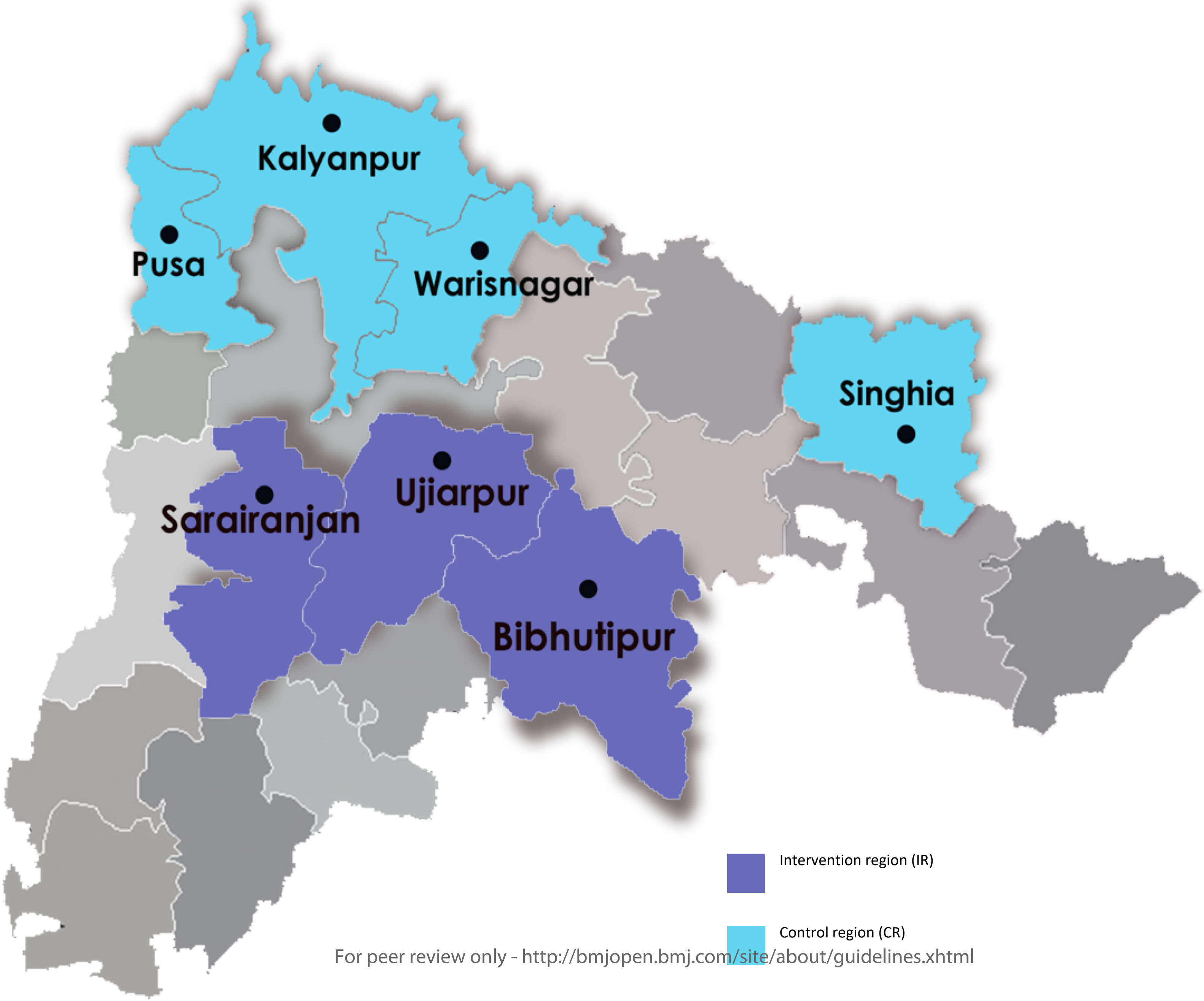

For peer review only - http://bmjopen.bmj.com/site/about/guidelines.xhtml

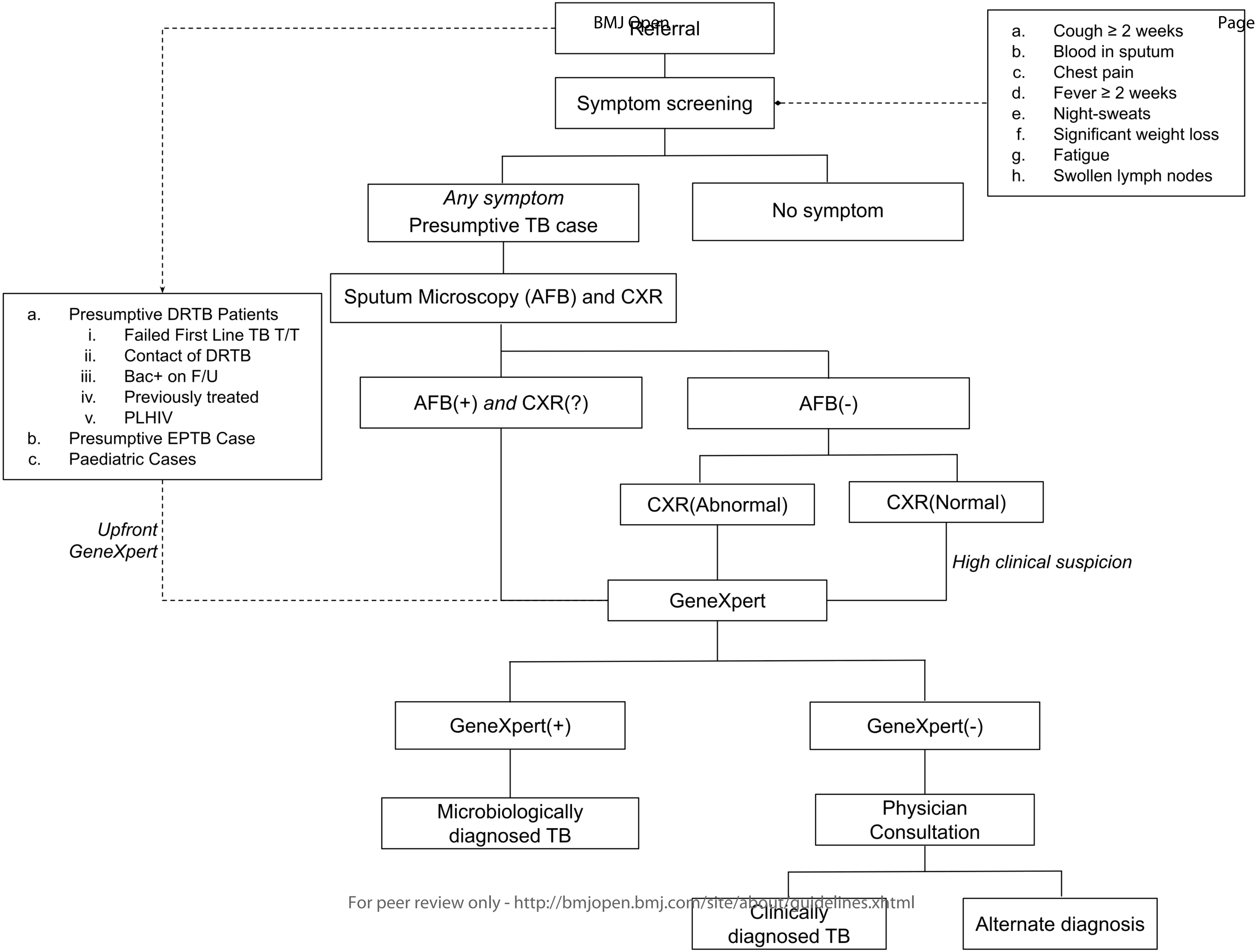

For peer review only - http://bmjopen.bmj.com/site/about/guidelines.xhtml

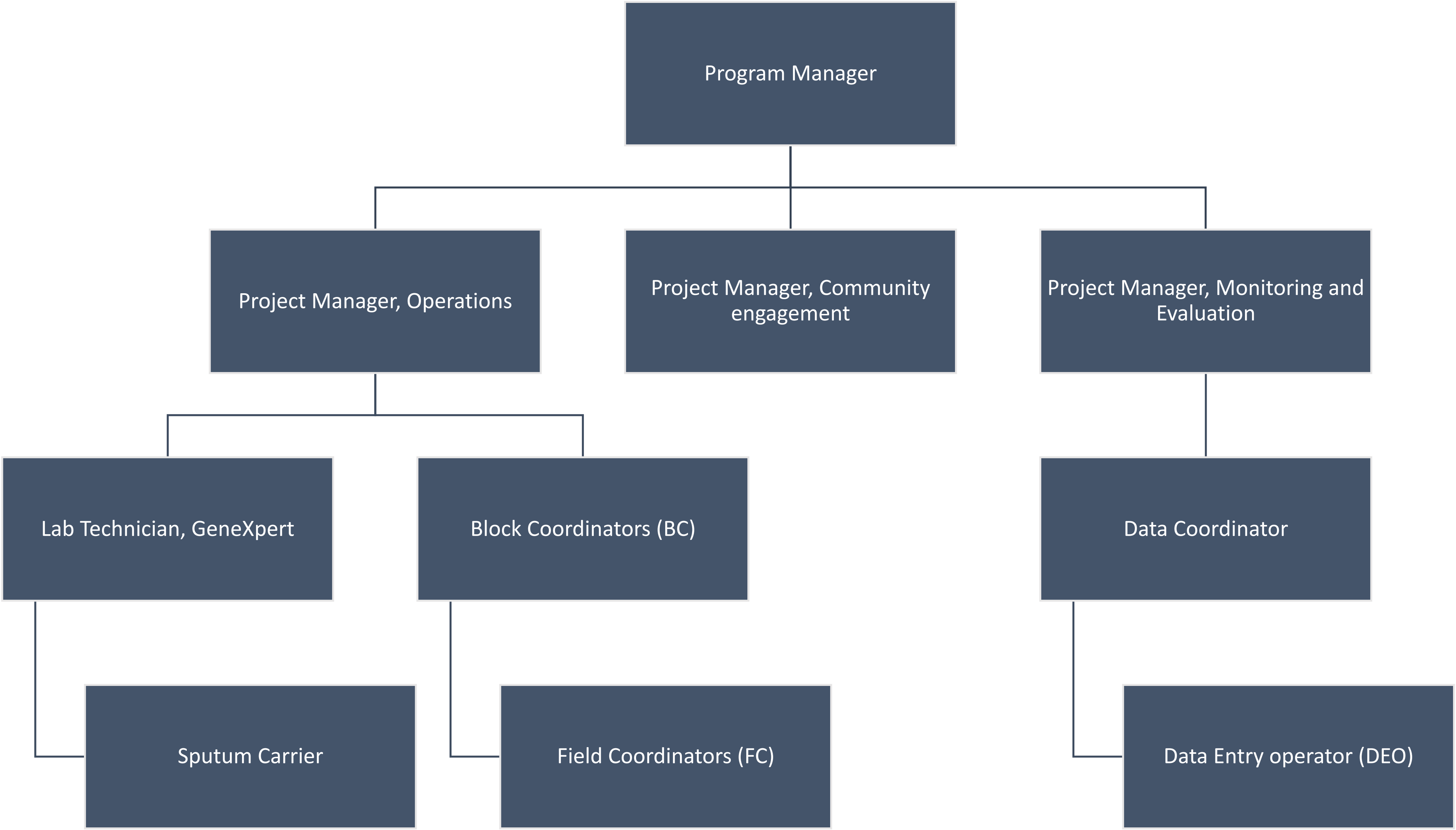
Program Manager
Project Manager, Operations
Project Manager, Community engagement
Project Manager, Monitoring and Evaluation
Lab Technician, GeneXpert
Block Coordinators (BC)
Data Coordinator
Sputum Carrier
Field Coordinators (FC)
Data Entry operator (DEO)

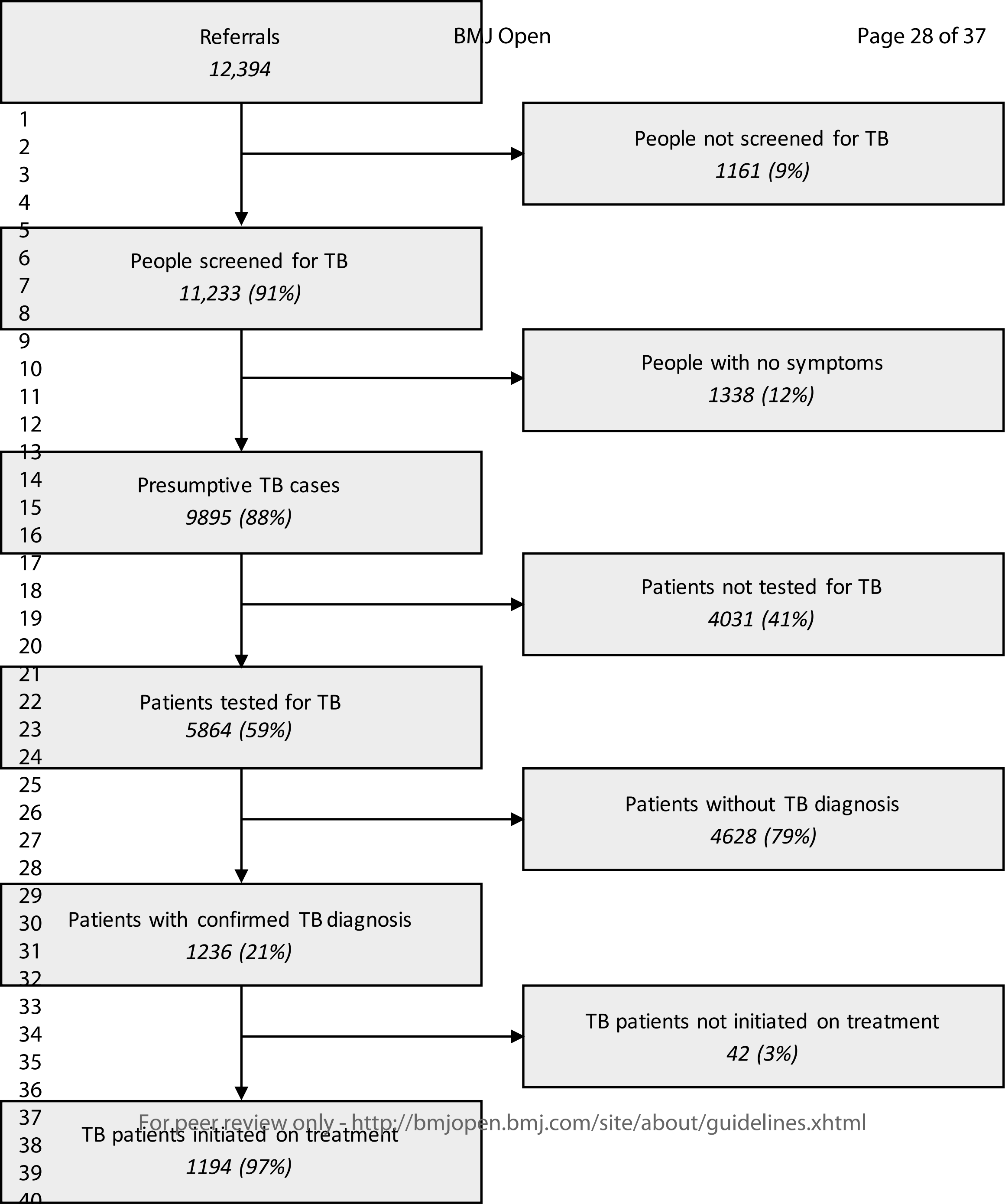

For peer review only - http://bmjopen.bmj.com/site/about/guidelines.xhtml

Supplementary File 1: The cost breakdown of the active case-finding program from Q3 2017 to Q2 2018 with cost allocation between case-finding and treatment support activities

									Cost allocation		Case-finding costs proportionate to the cost allocation				
#	Category	Item	2017Q3	2017Q4	2018Q1	2018Q2	Total	Comment	Case-finding	Treatment	C2017Q3	C2017Q4	C2018Q1	C2018Q2	Ctotal
1	Activities	Trainers, per-diem costs	₹ 29,850	₹ 29,850	₹ 29,850	₹ 29,850	₹ 1,19,400		100%	0%	₹ 29,850	₹ 29,850	₹ 29,850	₹ 29,850	₹ 1,19,400
2	Activities	Case-finding incentives	₹ 1,39,500	₹ 1,48,000	₹ 1,57,000	₹ 1,52,500	₹ 5,97,000	Unit cost INR500	100%	0%	₹ 1,39,500	₹ 1,48,000	₹ 1,57,000	₹ 1,52,500	₹ 5,97,000
3	Activities	Treatment completion incentives	₹ 2,65,050	₹ 2,81,200	₹ 2,98,300	₹ 2,89,750	₹ 11,34,300	Unit cost INR1000	0%	100%	₹ -	₹ -	₹ -	₹ -	₹ -
4	Activities	Field visits (fuel costs, management)	₹ 2,71,541	₹ 3,11,828	₹ 3,69,122	₹ 3,23,806	₹ 12,76,297		66%	34%	₹ 1,80,029	₹ 2,06,739	₹ 2,44,724	₹ 2,14,680	₹ 8,46,171
5	Activities	Training and workshops	₹ 65,395	₹ 52,488	₹ 58,690	₹ 2,69,343	₹ 4,45,916		55%	45%	₹ 35,925	₹ 28,835	₹ 32,242	₹ 1,47,965	₹ 2,44,967
6	Activities	Transport allowance	₹ 24,059	₹ 73,053	₹ 1,06,012	₹ 1,04,381	₹ 3,07,505		100%	0%	₹ 24,059	₹ 73,053	₹ 1,06,012	₹ 1,04,381	₹ 3,07,505
7	Activities	Communication material	₹ 24,474	₹ 15,400	₹ 50,265	₹ 16,950	₹ 1,07,089		100%	0%	₹ 24,474	₹ 15,400	₹ 50,265	₹ 16,950	₹ 1,07,089
8	Administrative overheads	Information technology (mobile data, voice)	₹ 25,020	₹ 25,638	₹ 16,969	₹ 13,150	₹ 80,777		66%	34%	₹ 16,588	₹ 16,998	₹ 11,250	₹ 8,718	₹ 53,554
9	Administrative overheads	Car rental (per month)	₹ 1,50,000	₹ 1,50,000	₹ 1,50,000	₹ 1,50,000	₹ 6,00,000		81%	19%	₹ 1,21,835	₹ 1,21,835	₹ 1,21,835	₹ 1,21,835	₹ 4,87,339
10	Administrative overheads	Rent	₹ 43,900	₹ 51,900	₹ 51,900	₹ 51,900	₹ 1,99,600		81%	19%	₹ 35,657	₹ 42,155	₹ 42,155	₹ 42,155	₹ 1,62,121
11	Administrative overheads	Electricity	₹ 4,085	₹ 8,055	₹ 7,740	₹ 7,654	₹ 27,534		81%	19%	₹ 3,318	₹ 6,543	₹ 6,287	₹ 6,217	₹ 22,364
12	Administrative overheads	Supplies (stationery, workshops, etc.)	₹ 1,93,094	₹ 1,10,390	₹ 88,210	₹ 78,000	₹ 4,69,694		81%	19%	₹ 1,56,837	₹ 89,662	₹ 71,647	₹ 63,354	₹ 3,81,500
13	Human resources	Program manager	₹ 2,10,000	₹ 2,10,000	₹ 2,10,000	₹ 2,10,000	₹ 8,40,000		81%	19%	₹ 1,70,569	₹ 1,70,569	₹ 1,70,569	₹ 1,70,569	₹ 6,82,275
14	Human resources	Project manager (community and training)	₹ 1,35,000	₹ 1,35,000	₹ 1,35,000	₹ 2,03,226	₹ 6,08,226		81%	19%	₹ 1,09,651	₹ 1,09,651	₹ 1,09,651	₹ 1,65,067	₹ 4,94,020
15	Human resources	Project manager (service delivery)	₹ 2,10,484	₹ 2,70,000	₹ 1,46,613	₹ 1,35,000	₹ 7,62,097		81%	19%	₹ 1,70,962	₹ 2,19,303	₹ 1,19,084	₹ 1,09,651	₹ 6,18,999
16	Human resources	GeneXpert technician	₹ 56,903	₹ 1,02,000	₹ 1,02,000	₹ 1,02,000	₹ 3,62,903		100%	0%	₹ 56,903	₹ 1,02,000	₹ 1,02,000	₹ 1,02,000	₹ 3,62,903
17	Human resources	MIS operator	₹ 49,484	₹ 90,250	₹ 1,01,000	₹ 1,16,250	₹ 3,56,984		66%	34%	₹ 32,807	₹ 59,835	₹ 66,962	₹ 77,073	₹ 2,36,677
18	Human resources	Consultants	₹ 1,66,833	₹ 1,65,000	₹ -	₹ -	₹ 3,31,833		100%	0%	₹ 1,66,833	₹ 1,65,000	₹ -	₹ -	₹ 3,31,833
19	Human resources	Block coordinators (BCs)	₹ 1,92,000	₹ 1,92,000	₹ 1,92,000	₹ 1,92,000	₹ 7,68,000		62%	38%	₹ 1,19,897	₹ 1,19,897	₹ 1,19,897	₹ 1,19,897	₹ 4,79,588
20	Human resources	Field coordinators (FCs)	₹ 4,11,196	₹ 4,02,155	₹ 4,48,906	₹ 4,59,767	₹ 17,22,024		55%	45%	₹ 2,25,893	₹ 2,20,926	₹ 2,46,609	₹ 2,52,576	₹ 9,46,005
21	Procurement of medical items	GeneXpert, test cartridge	₹ 1,29,006	₹ 6,26,923	₹ 6,20,888	₹ 3,39,489	₹ 17,16,306	Unit cost USD11.26	100%	0%	₹ 1,29,006	₹ 6,26,923	₹ 6,20,888	₹ 3,39,489	₹ 17,16,306
22	Procurement of medical items	Contingency, drugs	₹ 38,127	₹ 98,864	₹ 1,28,695	₹ 1,65,859	₹ 4,31,545		0%	100%	₹ -	₹ -	₹ -	₹ -	₹ -
23	Procurement of medical items	Contingency, chest X-rays	₹ 1,32,823	₹ 2,57,945	₹ 3,39,280	₹ 2,11,545	₹ 9,41,593		100%	0%	₹ 1,32,823	₹ 2,57,945	₹ 3,39,280	₹ 2,11,545	₹ 9,41,593
24	Procurement of medical items	Contingency, sputum microscopy	₹ 11,280	₹ 3,980	₹ 13,400	₹ 9,200	₹ 37,860		100%	0%	₹ 11,280	₹ 3,980	₹ 13,400	₹ 9,200	₹ 37,860
25	Procurement of medical items	Extra-pulmonary TB diagnostics	₹ 10,986	₹ 33,907	₹ 73,800	₹ 69,324	₹ 1,88,017		100%	0%	₹ 10,986	₹ 33,907	₹ 73,800	₹ 69,324	₹ 1,88,017
26	Procurement of medical items	Complications and hospitalization	₹ 25,719	₹ 45,597	₹ 42,386	₹ 36,111	₹ 1,49,813		0%	100%	₹ -	₹ -	₹ -	₹ -	₹ -
27	Procurement of medical items	Sputum containers (for transport)	₹ 8,894	₹ -	₹ -	₹ -	₹ 8,894		100%	0%	₹ 8,894	₹ -	₹ -	₹ -	₹ 8,894
28	Procurement of medical items	Customs duty and Xpert shipping	₹ 53,694	₹ 2,60,934	₹ 2,58,422	₹ 1,41,300	₹ 7,14,350	Unit cost INR314	100%	0%	₹ 53,694	₹ 2,60,934	₹ 2,58,422	₹ 1,41,300	₹ 7,14,350
29		*Additional information*													
30		Cartridges used	171	831	823	450									
31		Total TB diagnosed	284	302	324	326									
32		Total TB treatments started	279	296	314	305		See patient cascade in the figure							
33		Total treatment completed	265	281	298	290		Assumed at 95%							

* All cost figures are reported in Indian Rupee (INR or ₹)
* The cost allocation is sourced from the time calculations in Supplementary File 2
* The case-finding incentive (item 2) is derived from total cases diagnosed (item 31)
* The treatment completion incentive (item 3) is derived from total treatment completed (item 33)
* The GeneXpert costs (item 21 and 28) are derived from the actual consumption of the cartridges (item 30)

For peer review only

Supplementary File 2: The time allocation of human resource between case-finding and treatment support activities

Table A: Detailed time allocation for human resource

#	Human Resource	Activities	Value	Comment
1	**Field coordinator (FC)**	Work hours (in hours)	48	6 working days, 8 hours a day; 5 day field work + 1 day reporting-review-planning-training
		Total patients diagnosed per month	105	100 - 110 patients diagnosed per month
		Average treatment duration per patient (in months)	7	DSTB treatment duration of 6 - 8 months
		Patients per field coordinator, steady state	35	18 field coordinators in the program
		Travel per week (in hours)	193	4 weeks in a month. Average travel reimbursement per field coordinator of ₹2700 at ₹3.5 per km.
1.1		**Travel time per week (in hours)**	10	Average speed of 20 kmph
1.2		**Treatment support activities**		
		Time per visit (in hours)	1	Half hour on average
		Number of visits per patient in a month	3	3 times a month
		Total time spent per week in treatment support activities (in hours)	13	
1.3		**Case-finding activities**		
		Time spent in ASHA (CHW) training per week (in hours)	2	Average 1 meeting per week lasting nearly 2 hour
		Time spent in meeting individual ASHAs per week (in hours)	2	4 ASHAs per week, 0.5 hour per meeting
		Time spent in hospital visits and assisting in diagnosis per week (in hours)	6	3 times a week, 2 hours per visit
		Time spent in meeting referrals and screening per week (in hours)	6	12 per week, 0.5 hour per visit
		Total time spent per week in case-finding activities (in hours)	16	
1.4		**Reporting-review-planning-training, time spent per week (in hours)**	8	1 day
2	**Block coordinator (BC)**	Work hours (in hours)	48	6 working days, 8 hours a day
2.1		Admin-reporting-review-planning, time spent per week (in hours)	8	1 complete day, focused around FCs in their area
2.2		Block review-data management-project meeting-training, time spent per week (in hours)	8	Project meeting once a week, data management with MIS team, reviewing project performance, training
2.3		Supervisory work, time spent per week (in hours)	32	Split in proportion to FCs time spent in activities
3	**Managers**	Work hours	48	6 working days, 8 hours a day
3.1		Review-planning-design, time spent per week (in hours)	24	3 days a week, largely centered around case-finding
3.2		Supervisory work, time spent per week (in hours)	24	Split in proportion to BCs time spent in activities
4	**Data management team**	Work hours	48	Split in proportion of FCs time under reporting-review-planning

Table B: Summary of time allocation between case-finding and treatment support activities

Field coordinator (FC)	Activity	Time	Travel	Reporting-review-planning-training	Total	Proportion
1	Case-finding activities	16.0	5.3	4.4	25.7	55%
2	Treatment support activities	13.1	4.3	3.6	21.1	45%

Note: Time spent in travel and reporting-review-planning-training is proportionate to time spent in respective case-finding and treatment activities.

Block coordinator (BC)	Activity	Admin-reporting-review-planning	Block review-data management-project meeting-training	Supervisory work, time spent per week	Proportion
1	Case-finding activities	4.4	8.0	17.6	62%
2	Treatment support activities	3.6	0.0	14.4	38%

Managers	Activity	Review-planning-design	Supervisory work	Proportion
1	Case-finding activities	24	15.0	81%
2	Treatment support activities	0	9.0	19%

Data management team	Activity	Time	Proportion
1	Case-finding activities	94.7	66%
2	Treatment support activities	48.1	34%

Table C: Proportion of time allocation for human resource

#	Human Resource	Case-finding	Treatment support
1	Field coordinator (FC)	55%	45%
2	Block coordinator (BC)	62%	38%
3	Managers	81%	19%
4	Data management team	66%	34%

For peer review only

Supplementary File 3: Additional information on ASHA

Table A: Number of ASHAs

#	Block	Number of ASHA^	Population	ASHAs per 1000 pop.
1	Ujiarpur	264	341906	0.77
2	Saraianjan	236	287760	0.82
3	Bibhutipur	293	391817	0.75
	Total	793	1021483	0.78

Note: ^ Data as of December 2017.

Table B: Work of FC and ASHAs

Indicator	Steady state	Maximum
Total FC	18	23
Pop. per FC	56749	44412
ASHA per FC	44	34
Intervention pop.	1021483	

Note:

1. FC is Field Coordinator

2. Steady state inicates matured program operations and maximum indicate their highest value during study period.

For peer review only

Table C1: ASHA's performance on reproductive, maternal, and child health program indicators in Samastipur, Bihar

		Baseline period				Implementation period				Quarterly average	
Indicator	Region	2016Q3	2016Q4	2017Q1	2017Q2	2017Q3	2017Q4	2018Q1	2018Q2	Baseline	Implementation
Total number of pregnant women registered for ANC	IR	5947	5137	6330	6229	6415	5895	6593	6178	5911	6270
	CR	5951	5333	6340	6769	6387	6083	6648	6190	6098	6327
Number of Institutional Deliveries conducted (Including C-Sections)	IR	4592	4116	3913	3225	5011	4419	3694	3134	3962	4065
	CR	4045	3646	3809	2739	4482	4015	3487	2631	3560	3654
Number of Immunisation sessions where ASHAs were present	IR	2528	2531	2535	2606	2528	2550	2574	2566	2550	2555
	CR	2743	2728	2719	2673	2583	2626	2674	2672	2716	2639

Table C2: Detailed disaggregation of data in Table C1

		Baseline period				Implementation period				
Indicator	Region	2016Q3	2016Q4	2017Q1	2017Q2	2017Q3	2017Q4	2018Q1	2018Q2	Block
Total number of pregnant women registered for ANC	IR	2312	1788	2376	2278	2323	1832	2267	2296	Bibhutipur
	IR	1562	1510	1858	1980	1959	1899	2169	1993	Sarairanjan
	IR	2073	1839	2096	1971	2133	2164	2157	1889	Ujiarpur
	CR	2157	1837	2461	2413	2278	2040	2477	2095	Kalyanpur
	CR	802	659	904	792	929	891	911	804	Pusa
	CR	1433	1220	1341	1714	1645	1761	1512	1552	Singhia
	CR	1559	1617	1634	1850	1535	1391	1748	1739	Warisnagar
Number of Institutional Deliveries conducted (Including C-Sections)	IR	1562	1501	1396	1125	1710	1531	1354	1180	Bibhutipur
	IR	1676	1458	1392	1157	1827	1605	1326	1063	Sarairanjan
	IR	1354	1157	1125	943	1474	1283	1014	891	Ujiarpur
	CR	1059	931	1093	725	1160	867	938	801	Kalyanpur
	CR	816	675	661	546	833	716	630	502	Pusa
	CR	995	995	1033	671	1186	1264	986	639	Singhia
	CR	1175	1045	1022	797	1303	1168	933	689	Warisnagar
Number of Immunisation sessions where ASHAs were present	IR	923	909	915	933	931	907	927	928	Bibhutipur
	IR	736	765	763	800	768	767	772	773	Sarairanjan
	IR	869	857	857	873	829	876	875	865	Ujiarpur
	CR	995	956	976	982	969	964	980	971	Kalyanpur
	CR	424	445	412	388	363	363	373	378	Pusa
	CR	606	622	628	631	588	617	615	614	Singhia
	CR	718	705	703	672	663	682	706	709	Warisnagar

Note:
1.This is the dataset used for Table 3 in the manuscript.
2. Baseline period: Q3 of 2016 to Q2 of 2017
3. Study period: Q3 of 2017 to Q2 of 2018
4. IR: Intervention region
5. CR: Control region
6. ASHA: Accredited Social Health Activist
7. ANC: Antenatal checkup

For peer review only

Supplementary File 4: The disaggregated yield of the active case-finding program from Q3 2017 to Q2 2018.

Table A: Summary of yield by various blocks

#	Indicator	Total	Bibhutipur	Sarairanjan	Ujiarpur
1	# of people eligible for screening (referrals)	12394	4702	3764	3928
2	# of people screened	11233	4175	3403	3655
3	# of people with TB symptoms	9895	3573	3066	3256
4	# of people tested/evaluated for TB	5864	2062	1870	1932
5	# of people diagnosed with TB	1236	439	461	336
6	# of people initiated on treatment	1194	427	437	330
7	# of people diagnosed with TB (Bac+)	637	167	253	217
8	Proportion of microbiologically diagnosed cases	52%	38%	55%	65%
9	Pre-diagnostic loss to follow-up	41%	42%	39%	41%
10	Pre-treatment loss to follow-up	3%	3%	5%	2%
11	% screened of referred	91%	89%	90%	93%
12	% presumptive of screened	88%	86%	90%	89%
13	% tested of presumptive	59%	58%	61%	59%
14	% diagnosed with TB of tested	21%	21%	25%	17%

Note: Bac+ means microbiologically-confirmed TB.

For peer review only

Table B: Details of yield by various blocks and quarters

		Bibhutipur				Sarairanjan				Ujiarpur				Overall			
**#**	**Indicator**	**B 17Q3**	**B 17Q4**	**B 18Q1**	**B 18Q2**	**S 17Q3**	**S 17Q4**	**S 18Q1**	**S 8Q2**	**U 17Q3**	**U 17Q4**	**U 18Q1**	**U 18Q2**	**17Q3**	**17Q4**	**18Q1**	**18Q2**
1	# of people eligible for screening (referrals)	830	1320	1513	1039	794	781	988	1201	678	1080	1126	1044	2302	3181	3627	3284
2	# of people screened	698	1135	1420	922	648	737	933	1085	618	974	1078	985	1964	2846	3431	2992
3	# of people with TB symptoms	495	983	1273	822	593	662	870	941	502	894	983	877	1590	2539	3126	2640
4	# of people tested/evaluated for TB	251	566	762	483	334	383	574	579	276	519	597	540	861	1468	1933	1602
5	# of people diagnosed with TB	83	102	136	118	121	114	114	112	80	86	74	96	284	302	324	326
6	# of people initiated on treatment	83	100	132	112	117	111	109	100	79	85	73	93	279	296	314	305
7	# of people diagnosed with TB (Bac+)	32	45	53	37	50	51	78	74	55	48	53	61	137	144	184	172
8	Proportion of microbiologically diagnosed cases	39%	44%	39%	31%	41%	45%	68%	66%	69%	56%	72%	64%	48%	48%	57%	53%
9	Pre-diagnostic loss to follow-up	49%	42%	40%	41%	44%	42%	34%	38%	45%	42%	39%	38%	46%	42%	38%	39%
10	Pre-treatment loss to follow-up	0%	2%	3%	5%	3%	3%	4%	11%	1%	1%	1%	3%	2%	2%	3%	6%
11	% screened of referred	84%	86%	94%	89%	82%	94%	94%	90%	91%	90%	96%	94%	85%	89%	95%	91%
12	% presumptive of screened	71%	87%	90%	89%	92%	90%	93%	87%	81%	92%	91%	89%	81%	89%	91%	88%
13	% tested of presumptive	51%	58%	60%	59%	56%	58%	66%	62%	55%	58%	61%	62%	54%	58%	62%	61%
14	% diagnosed with TB of tested	33%	18%	18%	24%	36%	30%	20%	19%	29%	17%	12%	18%	33%	21%	17%	20%
		Bibhutipur				Sarairanjan				Ujiarpur				Overall			

Note:

1. B: Bibhutipur

2. S: Sarairanjan

3. U: Ujiarpur

For peer review only

CHEERS Checklist
Items to include when reporting economic evaluations of health interventions

The **ISPOR CHEERS Task Force Report**, *Consolidated Health Economic Evaluation Reporting Standards (CHEERS)—Explanation and Elaboration: A Report of the ISPOR Health Economic Evaluations Publication Guidelines Good Reporting Practices Task Force*, provides examples and further discussion of the 24-item CHEERS Checklist and the CHEERS Statement. It may be accessed via the *Value in Health* or via the ISPOR Health Economic Evaluation Publication Guidelines – CHEERS: Good Reporting Practices webpage: http://www.ispor.org/TaskForces/EconomicPubGuidelines.asp

Section/item	Item No	Recommendation	Reported on page No/ line No
**Title and abstract**			
Title	1	Identify the study as an economic evaluation or use more specific terms such as "cost-effectiveness analysis", and describe the interventions compared.	1
Abstract	2	Provide a structured summary of objectives, perspective, setting, methods (including study design and inputs), results (including base case and uncertainty analyses), and conclusions.	2
**Introduction**			
Background and objectives	3	Provide an explicit statement of the broader context for the study. Present the study question and its relevance for health policy or practice decisions.	4
**Methods**			
Target population and subgroups	4	Describe characteristics of the base case population and subgroups analysed, including why they were chosen.	5
Setting and location	5	State relevant aspects of the system(s) in which the decision(s) need(s) to be made.	5
Study perspective	6	Describe the perspective of the study and relate this to the costs being evaluated.	7
Comparators	7	Describe the interventions or strategies being compared and state why they were chosen.	5 5
Time horizon	8	State the time horizon(s) over which costs and consequences are being evaluated and say why appropriate.	5 4
Discount rate	9	Report the choice of discount rate(s) used for costs and outcomes and say why appropriate.	Not applicable
Choice of health outcomes	10	Describe what outcomes were used as the measure(s) of benefit in the evaluation and their relevance for the type of analysis performed.	7, 8
Measurement of effectiveness	11a	*Single study-based estimates:* Describe fully the design features of the single effectiveness study and why the single study was a sufficient source of clinical effectiveness data.	Not applicable

For peer review only

	11b	*Synthesis-based estimates:* Describe fully the methods used for identification of included studies and synthesis of clinical effectiveness data.	Not applicable
Measurement and valuation of preference based outcomes	12	If applicable, describe the population and methods used to elicit preferences for outcomes.	Not applicable
Estimating resources and costs	13a	*Single study-based economic evaluation:* Describe approaches used to estimate resource use associated with the alternative interventions. Describe primary or secondary research methods for valuing each resource item in terms of its unit cost. Describe any adjustments made to approximate to opportunity costs.	8
	13b	*Model-based economic evaluation:* Describe approaches and data sources used to estimate resource use associated with model health states. Describe primary or secondary research methods for valuing each resource item in terms of its unit cost. Describe any adjustments made to approximate to opportunity costs.	Not applicable
Currency, price date, and conversion	14	Report the dates of the estimated resource quantities and unit costs. Describe methods for adjusting estimated unit costs to the year of reported costs if necessary. Describe methods for converting costs into a common currency base and the exchange rate.	9, 11
Choice of model	15	Describe and give reasons for the specific type of decision-analytical model used. Providing a figure to show model structure is strongly recommended.	Not applicable
Assumptions	16	Describe all structural or other assumptions underpinning the decision-analytical model.	Not applicable
Analytical methods	17	Describe all analytical methods supporting the evaluation. This could include methods for dealing with skewed, missing, or censored data; extrapolation methods; methods for pooling data; approaches to validate or make adjustments (such as half cycle corrections) to a model; and methods for handling population heterogeneity and uncertainty.	8, 9
**Results**			
Study parameters	18	Report the values, ranges, references, and, if used, probability distributions for all parameters. Report reasons or sources for distributions used to represent uncertainty where appropriate. Providing a table to show the input values is strongly recommended.	10
Incremental costs and outcomes	19	For each intervention, report mean values for the main categories of estimated costs and outcomes of interest, as well as mean differences between the comparator groups. If applicable, report incremental cost-effectiveness ratios.	Not applicable
Characterising uncertainty	20a	*Single study-based economic evaluation:* Describe the effects of sampling uncertainty for the estimated incremental cost and incremental effectiveness parameters, together with the impact	Not applicable

For peer review only

		of methodological assumptions (such as discount rate, study perspective).	
	20b	*Model-based economic evaluation:* Describe the effects on the results of uncertainty for all input parameters, and uncertainty related to the structure of the model and assumptions.	Not applicable
Characterising heterogeneity	21	If applicable, report differences in costs, outcomes, or cost-effectiveness that can be explained by variations between subgroups of patients with different baseline characteristics or other observed variability in effects that are not reducible by more information.	Not applicable
**Discussion**			
Study findings, limitations, generalisability, and current knowledge	22	Summarise key study findings and describe how they support the conclusions reached. Discuss limitations and the generalisability of the findings and how the findings fit with current knowledge.	12, 13
**Other**			
Source of funding	23	Describe how the study was funded and the role of the funder in the identification, design, conduct, and reporting of the analysis. Describe other non-monetary sources of support.	17
Conflicts of interest	24	Describe any potential for conflict of interest of study contributors in accordance with journal policy. In the absence of a journal policy, we recommend authors comply with International Committee of Medical Journal Editors recommendations.	17

For consistency, the CHEERS Statement checklist format is based on the format of the CONSORT statement checklist

The **ISPOR CHEERS Task Force Report** provides examples and further discussion of the 24-item CHEERS Checklist and the CHEERS Statement. It may be accessed via the *Value in Health* link or via the ISPOR Health Economic Evaluation Publication Guidelines – CHEERS: Good Reporting Practices webpage: http://www.ispor.org/TaskForces/EconomicPubGuidelines.asp

The citation for the CHEERS Task Force Report is:

Husereau D, Drummond M, Petrou S, et al. Consolidated health economic evaluation reporting standards (CHEERS)—Explanation and elaboration: A report of the ISPOR health economic evaluations publication guidelines good reporting practices task force. Value Health 2013;16:231-50.

For peer review only
